# Identification of *ZmP5CS* Gene Family and Functional Analysis of *ZmP5CS4* Under Salt Tolerance in Maize

**DOI:** 10.3390/plants15060946

**Published:** 2026-03-19

**Authors:** Chunxiao Zhang, Liquan Sun, Jia Guo, Jing Dong, Fengxue Jin, Xiaohui Zhou, Xueyan Liu, Chengyuan Liu, Xiaohui Li

**Affiliations:** 1 Maize Research Institute, Jilin Academy of Agricultural Sciences (Northeast Agricultural Research Center of China), Gongzhuling 136100, China; cxzhang@jaas.com.cn (C.Z.); s1123691@163.com (L.S.); jdong@jaas.com.cn (J.D.); fxjin@jaas.com.cn (F.J.); xhzhou@jaas.com.cn (X.Z.); xyliu@jaas.com.cn (X.L.); 2 Agricultural College, Yanbian University, Yanji 133002, China; 3 Institute of Agriculture Biotechnology, Jilin Academy of Agricultural Sciences (Northeast Agricultural Research Center of China), Changchun 130033, China; grammy1981@163.com; 4 Gongzhuling Jinong Green Agriculture Hi-Tech Development Co., Ltd., Gongzhuling 136100, China

**Keywords:** maize, salt tolerance, *P5CS* gene family, association analysis of candidate genes, functional validation

## Abstract

Saline–alkali land represents an important reserve of arable resources in China, and exploiting its agricultural potential is crucial for ensuring food security. In maize (*Zea mays* L.), which is moderately sensitive to salt stress, proline serves as a key osmoprotectant, and Δ^1^-pyrroline-5-carboxylate synthetase (P5CS), the rate-limiting enzyme in its biosynthesis, plays a vital role in plant stress responses. In this study, the maize *ZmP5CS* gene family was systematically identified and characterized through comprehensive bioinformatics analyses. Four *ZmP5CS* homologs were identified, most of which were predicted to localize to chloroplasts. Phylogenetic analysis classified these genes into four major clades. Among them, *ZmP5CS4* (*GRMZM2G028535*) expression was significantly upregulated under salt stress. Association analysis using a natural population of 278 inbred lines revealed that nine SNPs significantly associated with relative P5CS enzyme activity were located within *ZmP5CS4*. Haplotype analysis further identified a superior haplotype, HapA, carried by 14 inbred lines. Under salt stress, lines carried by HapA exhibited higher P5CS enzyme activity, greater proline accumulation, lower standard evaluation scores, and slightly enhanced salt tolerance compared to lines carried by HapB. Functional validation via transgenic approaches demonstrated that *ZmP5CS4* overexpression significantly increased proline content and plant survival under salt stress, whereas knockout of this gene led to heightened salt sensitivity. Collectively, this study elucidates the structure and function of the maize *ZmP5CS* gene family, establishes the critical role of *ZmP5CS4* in the salt stress response, and provides both a theoretical foundation and a candidate gene resource for improving salt tolerance in maize breeding programs.

## 1. Introduction

Saline–alkali land constitutes a vital strategic reserve of cultivated land resources in China, with profound implications for safeguarding national food security and expanding arable land availability. Maximal exploitation of its agricultural potential has therefore become a strategic priority [1]. China currently contains approximately 3.67 × 10^7^ hm^2^ of utilizable saline–alkali land, of which about 6.67 × 10^6^ hm^2^ (including 2.00 × 10^6^ hm^2^ located in the northeastern saline–alkali region [2]) is considered to possess agricultural development value and near-term potential for reclamation. Compared to other major cereals such as wheat and rice, maize is moderately sensitive to salt stress, which has become a major factor limiting its productivity [3,4]. Consequently, identifying superior resistance genes and developing a novel maize germplasm combining salt–alkali tolerance with desirable agronomic traits represent viable strategies for enhancing grain production on these marginal lands.

Under salt stress, higher plants primarily regulate their osmotic potential by accumulating inorganic ions and synthesizing compatible solutes [5,6,7,8]. However, reliance on inorganic ions for osmotic adjustment does not fully mitigate the damage caused by osmotic stress. Sustained accumulation of inorganic ions can lead to toxic ion concentrations, resulting in secondary injury to plants [9]. Among the various osmolytes, proline has attracted considerable research attention due to its widespread accumulation in plants under abiotic stress. As a compatible solute, proline not only stabilizes protein structures, but also regulates cellular redox potential [10,11]. Proline biosynthesis occurs via two pathways: the glutamate (Glu) pathway and the ornithine (Orn) pathway [12,13]. In the glutamate pathway, glutamate is reduced to glutamic-γ-semialdehyde (GSA) by Δ^1^-pyrroline-5-carboxylate synthetase (P5CS), which spontaneously cyclizes to pyrroline-5-carboxylate (P5C). This process typically takes place in the cytosol and chloroplasts [14]. P5CS is a key enzyme in plant proline biosynthesis, catalyzing the rate-limiting step in the glutamate pathway and thereby regulating proline content [14]. Enhanced P5CS activity can stimulate proline accumulation, consequently improving the osmotic adjustment capacity of plants under environmental stress [15,16].

Given its regulatory role in proline biosynthesis, P5CS function has been extensively investigated across multiple species [17,18]. Heterologous expression of a mothbean *P5CS* gene in rice was shown to increase proline content 10–18-fold, accompanied by significantly enhanced salinity tolerance and root biomass [19]. Subsequent studies in various species, including tobacco, wheat, carrot, sugarcane, and rice, have further demonstrated that *P5CS* overexpression significantly elevates proline levels and is closely associated with improved salt stress tolerance [20,21,22,23]. In addition to salinity tolerance, *P5CS* overexpression has been shown to markedly improve drought tolerance in plants. Under drought stress, such transgenic plants commonly exhibit higher survival rates, delayed wilting, increased biomass, enhanced osmotic adjustment and antioxidant capacity, and improved photosynthetic rates and yields. These positive effects have been confirmed in a range of crops, including rice, wheat, chickpea, tobacco, petunia, and citrus [19,24,25,26,27,28,29,30,31,32,33]. Conversely, suppression of *P5CS* expression results in increased stress sensitivity. Arabidopsis mutants with weakened *P5CS* function show reduced proline accumulation, elevated ROS-mediated damage, and diminished antioxidant enzyme activity [34,35]. Similarly, antisense suppression of *P5CS* in soybean led to significantly greater damage under drought stress compared to wild-type plants [17,18]. Despite extensive research on *P5CS* function in other species, the structural characteristics, evolutionary relationships, and functional divergence among maize *ZmP5CS* family members remain to be systematically characterized. Furthermore, the specific regulatory mechanism of maize *ZmP5CS* genes under salt stress and their potential as breeding targets have not been fully elucidated.

In this study, an integrated approach combining bioinformatics, population genetics, and transgenic functional validation was employed to systematically identify members of the *P5CS* gene family in maize. Their evolutionary relationships, structural characteristics, and expression patterns were thoroughly analyzed, and based on this foundation, superior haplotypes were screened through association analysis of a natural population. Furthermore, the role of *ZmP5CS4* in salt tolerance was functionally characterized by creating and analyzing both overexpression and knockout materials. This research aims to elucidate the mechanistic role of the *P5CS* gene family in the maize salt-stress response, thereby providing a theoretical basis and genetic resources for genetically improving salt tolerance in maize.

## 2. Results

### 2.1. Screening, Identification, and Characteristic Analysis of P5CS Gene Family Members

Four *P5CS* homologous genes were identified in the maize genome through a local BLASTp search and PFAM domain analysis, using the amino acid sequences of two known Arabidopsis *P5CS* proteins as queries. The molecular characteristics of the *P5CS* gene family in Arabidopsis, maize, rice, and sorghum are summarized in Table 1. The protein encoded by *GRMZM2G389416* (*ZmP5CS1*) comprises 362 amino acids, exhibits high predicted instability, and has a positively shifted isoelectric point. Although the protein encoded by *GRMZM2G061777* (*ZmPCS2*) is only 117 amino acids in length, it has been annotated as ‘pcs1-pyrroline-5-carboxylate synthase1’ in MaizeGDB. Additionally, the study by Walley et al. (2016) reported that *ZmPCS2* maintains a low expression level throughout the growth period of maize [36], providing transcript evidence for *ZmPCS2*. Typical full-length P5CS enzymes, which include maize *GRMZM2G375504* (*ZmP5CS3*) and *ZmP5CS4*; rice *Os01t0848200* and *Os05t0455500*; sorghum *SORBI_3003G356000* and *SORBI_3009G160100*; and Arabidopsis *AT2G39800* and *AT3G55610*, range from 716 to 735 amino acids in length, with molecular weights of approximately 77–79 kDa and theoretical isoelectric points (pI) between 5.9 and 6.4. This high degree of sequence conservation suggests strong evolutionary selection on the catalytic domains and that these proteins possess core enzymatic functions. Subcellular localization predictions using WoLF PSORT and CELLO v.2.5 software indicated that most *P5CS* family members are likely localized to chloroplasts, consistent with the known role of photosynthetic organelles in proline synthesis. However, Arabidopsis *AT3G55610* was predicted to localize to the endoplasmic reticulum, while maize *ZmP5CS4* and sorghum *SORBI_3003G356000* were predicted to reside in the plasma membrane. This differential localization suggests that distinct *P5CS* isoenzymes may contribute to proline biosynthesis in separate cellular compartments or under specific physiological conditions, potentially enabling spatially coordinated responses to stress or developmental signals.

### 2.2. Phylogenetic Analysis

**Figure 1:**
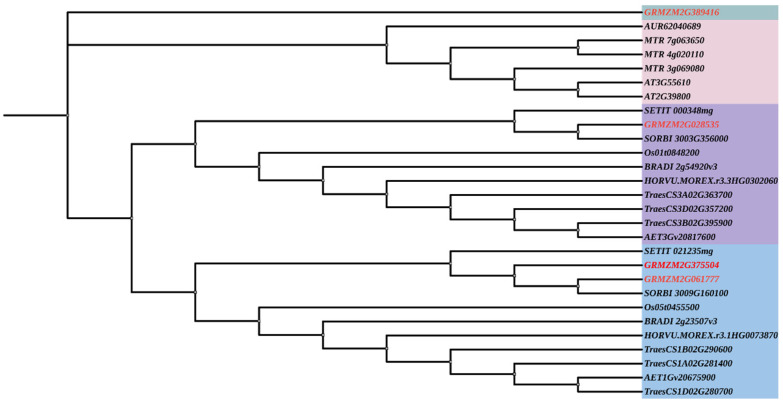
Phylogenetic analysis of *P5CS* gene families in 12 crop species. Different colors of shadow represent different clades, and the red font indicates members of the maize *P5CS* gene family.

A phylogenetic tree was constructed using *P5CS* protein sequences from a range of dicot and monocot plants to elucidate the evolutionary relationships among maize *ZmP5CS* genes (Figure 1). The analysis revealed that the *P5CS* family can be divided into four major clades, a branching pattern likely shaped by three key duplication events during its evolution.

Clade I exclusively contains maize *P5CS1*, which occupies a phylogenetically isolated position. This placement suggests a distinct evolutionary trajectory within the maize lineage, resulting in sequence features that diverge substantially from other homologs. Clade II consists primarily of two Arabidopsis *P5CS* genes (*AT2G39800* and *AT3G55610*) along with several legume members, representing the core dicot *P5CS* lineage. In contrast, Clades III and IV are predominantly composed of grass-specific P5CS genes. This distribution suggests a lineage-specific duplication event in the common ancestor of grasses, which may have provided the genetic basis for regulatory diversification within the proline synthesis pathway.

The four maize *ZmP5CS* genes exhibit distinct evolutionary origins based on their phylogenetic placement. *ZmP5CS1*, located in the isolated Clade I, possesses unique molecular characteristics—including sequence truncation and high predicted instability—consistent with its phylogenetic divergence. *ZmP5CS2* and *ZmP5CS3* both cluster within Clade IV, indicating that they originated from a single duplication event in the grass ancestor followed by functional divergence. *ZmP5CS3* retains a complete catalytic domain and a chloroplast-targeting signal, suggesting a primary role in basal proline synthesis. The truncated *P5CS2* protein may have evolved as a regulatory factor involved in fine-tuning proline metabolism.

*ZmP5CS4* groups within Clade III, a grass-specific clade evolutionarily distinct from Clade IV. Notably, *ZmP5CS4* is uniquely predicted to localize to the plasma membrane. An early duplication event in grass evolution is inferred to have given rise to separate Clade III and IV lineages, which subsequently underwent functional specialization.

### 2.3. Analysis of Cis-Acting Elements

**Figure 2:**
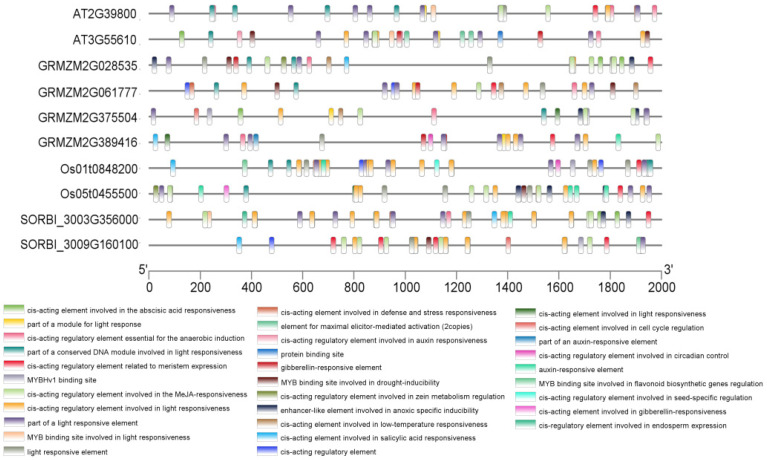
Analysis of cis-acting regulatory elements in the *P5CS* gene promoter region of Arabidopsis, maize, rice, and sorghum.

Cis-acting elements in the promoter regions (2 kb upstream of the ATG start codon) of the four maize *ZmP5CS* genes were analyzed to investigate their transcriptional regulation (Figure 2). All promoters were found to contain abscisic acid-responsive elements (ABREs) and methyl jasmonate-responsive elements (CGTCA-motif/TGACG-motif), indicating that their expression is likely co-modulated by the ABA and JA signaling pathways. This common regulatory feature provides a transcriptional basis for the rapid induction of proline synthesis under salt stress.

Further analysis revealed distinct cis-element compositions among the promoters, suggesting divergent regulatory roles. The promoter of the chloroplast-localized *ZmP5CS3* contained core stress-responsive elements as well as defense- and stress-related motifs (e.g., TC-rich repeats), which may contribute to sustained expression under prolonged stress. In contrast, the promoter of the truncated variant *ZmP5CS2* displayed a simpler architecture, consistent with a more basal regulatory mode. The *ZmP5CS1* promoter was notably enriched in MYB-binding sites (e.g., MBS), consistent with its rapid and strong stress-induced expression observed in preliminary analyses, suggesting a role as an early response regulator. The promoter of *ZmP5CS4*, which is predicted to localize to the plasma membrane, contained common stress-responsive elements, such as ABREs, and uniquely harbored an anaerobic response element (ARE). This implies that *ZmP5CS4* may respond not only to osmotic stress but also to hypoxia, potentially enabling localized proline synthesis near the plasma membrane.

Collectively, these findings indicate that while maize *ZmP5CS* genes share fundamental stress-responsive cis-elements, each member possesses distinct regulatory signatures. These specialized promoter features likely indicate differentiated functions—such as rapid signal perception, inducible activation, and sustained synthesis—thereby forming a coordinated yet functionally partitioned transcriptional network under stress conditions.

### 2.4. Analysis of Conserved Motifs, Domains, and Gene Structures

**Figure 3:**
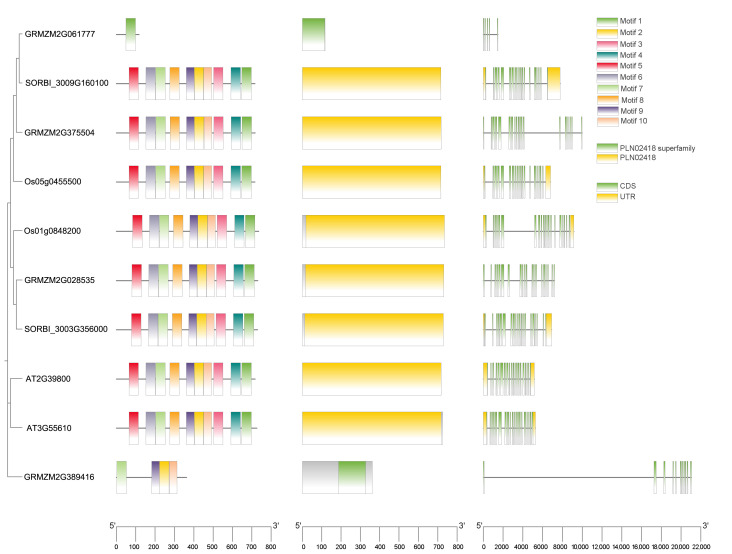
Sequence analysis of conserved bases of *P5CS* gene family members of Arabidopsis, maize, rice and sorghum.

To elucidate the structural characteristics of the maize *P5CS* gene family, an integrated analysis of conserved motifs, domain architecture, and gene structure (exon–intron organization) was performed (Figure 3). The results revealed pronounced differences in these features among members from different evolutionary clades, which were consistent with their phylogenetic relationships.

In the distribution of conserved motifs, typical members encoding full-length *P5CS* enzymes—such as maize *ZmP5CS3*, rice *Os05g0455500*, and Arabidopsis *AT2G39800*—contained a complete set of motifs (Motif 1–8). These motifs correspond to key catalytic domains, including GK, GSA ATPase, and GSA DH, reflecting strong evolutionary conservation of enzymatic function. *ZmP5CS4* (Clade III) showed high motif similarity to these typical genes and retained most core catalytic motifs, suggesting that it likely encodes a functional proline synthase.

In contrast, the two truncated maize genes exhibited substantial motif loss. *ZmP5CS2* (Clade IV) contained only a few motifs, consistent with its short coding sequence, whereas *ZmP5CS1* (Clade I) displayed the most distinct motif profile. It lacked many catalysis-related motifs but contained several unique ones, indicating potential evolution toward specialized regulatory roles.

At the gene structure level, members of Clade III and Clade IV—which originated from ancestral gene duplications in Poaceae—generally contained a relatively high number of introns and shared similar intron–exon patterns, indicating conserved structural organization. Among these, the gene structure of *ZmP5CS4* closely resembled those of its orthologs in rice and sorghum within the same clade, indicating stable genetic architecture across species. In contrast, *ZmP5CS1* exhibited a highly simplified gene structure distinct from other family members. Meanwhile, *ZmP5CS2* retained the structural framework characteristic of Clade IV but showed pronounced reduction in CDS length, suggesting that truncated transcripts may arise from alternative splicing or transcriptional variation.

Together, phylogenetic, motif, and gene structural evidence indicate divergent evolutionary paths for the truncated maize members. *ZmP5CS2* appears to have evolved within the conserved Clade IV framework into a stable, small, soluble protein, potentially acting as an auxiliary component in regulatory complexes. *ZmP5CS1* has independently evolved into a highly unstable basic protein, likely playing a role in rapid stress-response regulation. In contrast, the structurally intact and motif-conserved *ZmP5CS3* and *ZmP5CS4*, with stable gene architectures and complete catalytic motifs, are positioned to mediate proline biosynthesis under major abiotic stresses.

### 2.5. Chromosomal Localization and Collinearity Analysis

**Figure 4:**
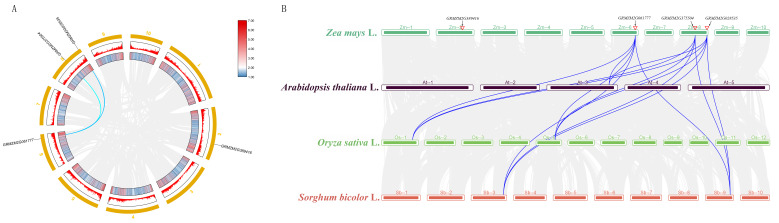
*P5CS* gene duplication and collinear correlations between maize and related species. (**A**) Duplicated *P5CS* genes in maize. (**B**) Collinear correlations of the *P5CS* genes between maize, arabidopsis, rice, and sorghum. Gene pairs with segmental duplication are linked by lines.

The chromosomal locations of the four maize *P5CS* gene family members were determined (Figure 4A), and they were found to be distributed across three chromosomes: *ZmP5CS1* on Chr.2, *ZmP5CS2* on Chr.6, and both *ZmP5CS3* and *ZmP5CS4* on Chr.8. This dispersed genomic distribution suggests independent origins via distinct duplication or rearrangement events.

Collinearity analysis was performed to trace the evolutionary history of these genes. Intraspecific analysis in maize revealed clear collinear blocks linking *ZmP5CS2*, *ZmP5CS3*, and *ZmP5CS4* (Figure 4), indicating that these three genes likely originated from a maize-specific whole-genome duplication event, followed by functional differentiation. In contrast, *ZmP5CS1* showed no collinearity with other family members. Given its distinct sequence and structural features, we propose that *ZmP5CS1* may have arisen through retrotransposon-mediated duplication or another lineage-specific mechanism.

Interspecies collinearity analysis further elucidated their evolutionary relationships. *ZmP5CS4* exhibited conserved synteny with rice *Os01t0848200* and sorghum *SORBI_3003G356000*, all belonging to Clade III. Similarly, *ZmP5CS3* showed clear collinearity with corresponding genes in rice and sorghum within Clade IV. These results strongly suggest an ancient duplication event in the common ancestor of Poaceae that gave rise to the separate Clade III and Clade IV lineages. Neither *ZmP5CS4* nor *ZmP5CS3* showed direct collinearity with *P5CS* genes from Arabidopsis thaliana, consistent with the phylogenetic divergence between monocot and dicot *P5CS* lineages early in plant evolution.

In summary, collinearity analyses delineate a multi-layered evolutionary framework: an ancient divergence established the monocot–dicot separation of *P5CS* genes; a lineage-specific duplication in the ancestral grass genome generated Clades III and IV; and subsequent whole-genome duplication, along with potential specialized mechanisms, shaped the four functionally distinct *P5CS* genes in maize. This evolutionary trajectory—from ancient duplication to recent diversification—provides genomic structural insight into the functional specialization of the proline synthesis regulatory network in maize.

### 2.6. Protein Interaction Network Analysis and Secondary-Structure Prediction

**Figure 5:**
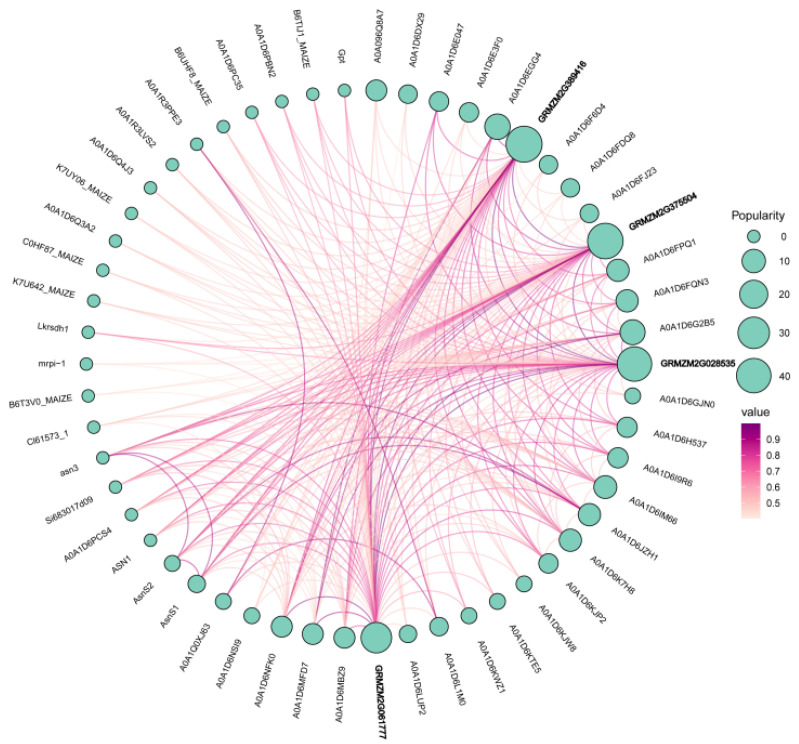
Protein interaction network of *ZmP5CS* gene family in maize.

Protein interaction network analysis revealed that all four maize *P5CS* members exhibit high-confidence direct interactions (confidence score: 0.817) with glutamate synthase 1 (NADH-dependent, chloroplastic) (Figure 5), suggesting functional coupling between *P5CS* and glutamate synthase to enhance substrate utilization and promote proline synthesis. Indirect interactions were also observed with enzymes involved in nitrogen metabolism, such as asparagine synthase and lysine ketoglutarate reductase, indicating integration of the proline synthesis pathway into a broader carbon–nitrogen metabolic network.

Protein secondary structure predictions showed distinct profiles between canonical and truncated *P5CS* members (Table 2). The full-length enzymes *ZmP5CS3* and *ZmP5CS4* contained a high proportion of α helices (48–50%) and moderate disordered regions (25–27%), consistent with stable catalytic structures. In contrast, the truncated members *ZmP5CS2* and *ZmP5CS1* displayed markedly higher disorder (38.46% and 40.06%, respectively) and lower α helix content. Elevated structural disorder is often associated with conformational flexibility, suggesting that these proteins may engage in dynamic molecular interactions or regulatory processes.

Taken together, these structural and interactomic data support a proposed functional division within the maize *P5CS* family: *ZmP5CS3* and *ZmP5CS4* likely serve as stable catalytic cores for basal proline synthesis, whereas the more flexible *ZmP5CS2* and *ZmP5CS1* may play regulatory roles, potentially modulating enzyme activity or metabolic flux through protein–protein interactions. These interactions are computational predictions based on homology and should be interpreted cautiously, particularly for truncated proteins. This structure–function hypothesis provides a framework for further experimental validation of intra-family interaction mechanisms and their effects on pathway regulation.

### 2.7. Gene Expression Validation

**Figure 6:**
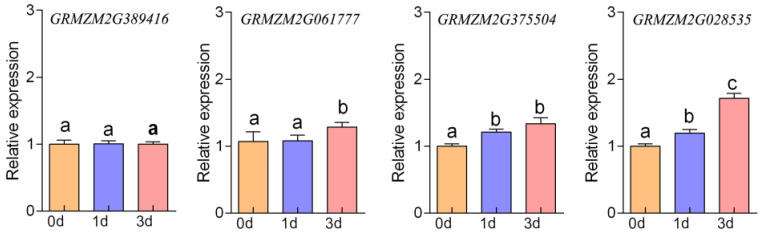
Differential expression of *P5CS* gene family in maize under salt stress. Different lower-case letters indicate statistically significant differences based on an ANOVA followed by Tukey’s HSD (*p* < 0.05).

The expression dynamics of four maize *P5CS* genes (*ZmP5CS1*, *ZmP5CS2*, *ZmP5CS3*, and *ZmP5CS4*) in leaves were analyzed by qRT-PCR at 0, 1, and 3 days after salt stress (Figure 6). Distinct temporal expression patterns were observed among family members, suggesting that they may function synergistically through differential transcriptional regulation in response to salt stress.

No significant change in *ZmP5CS1* expression was detected following stress. *ZmP5CS2*, which encodes a truncated protein, exhibited delayed upregulation: its expression on day 1 did not differ significantly from that of the control, but increased to 1.20 times that of the control by day 3 (*p* < 0.01). This pattern suggests that it may operate primarily at the post-translational level, possibly through protein–protein interactions.

The chloroplast-localized *ZmP5CS3* exhibited a steadily increasing expression profile. At 1 day post-stress, its expression was 1.21 times higher than that of the control (*p* < 0.01), and further increased to 1.10 times that of the 1-day level by day 3 (*p* < 0.05). These results indicate that *ZmP5CS3* may be involved in maintaining long-term proline homeostasis within chloroplasts.

*ZmP5CS4* showed sustained upregulation, with its expression reaching 1.19 and 1.72 times that of the control at 1 and 3 days, respectively (*p* < 0.01). This rapid and persistent induction is consistent with the stress-related cis elements identified in its promoter, indicating a role for *ZmP5CS4* in early stress perception and initiation of proline synthesis.

Collectively, these expression profiles indicate that maize *ZmP5CS* gene family members are differentially regulated over time under salt stress, likely enabling a coordinated transcriptional response across the proline biosynthesis pathway.

### 2.8. Association Analysis of Allelic Variation in ZmP5CS Genes and Relative P5CS Enzyme Activity

**Figure 7:**
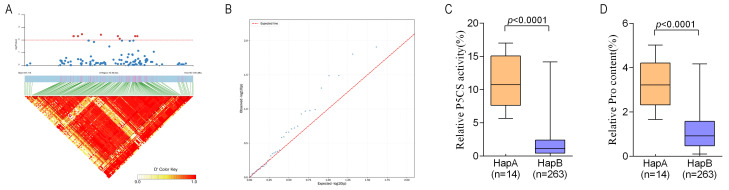
Candidate gene association analysis of *ZmP5CS4*. (**A**) Genome-wide association analysis of relative P5CS enzyme activity in 278 maize inbred lines. (**B**) Quantile–Quantile Plot. (**C**,**D**) Correlation analysis between different haplotypes and both relative P5CS activity and relative proline content.

To identify genetic loci regulating salt tolerance through the four maize *P5CS* genes, SNP information for each gene and its ~2 kb promoter region was extracted from public resequencing data. A total of 13, 63, 47, and 164 SNPs were identified in *ZmP5CS1*, *ZmP5CS2*, *ZmP5CS3*, and *ZmP5CS4*, respectively. Association analysis between these SNPs and relative *ZmP5CS* enzyme activity was performed across a panel of 278 maize inbred lines (Figure 7A,B). No significant associations were detected for SNPs within *ZmP5CS1*, *ZmP5CS2*, or *ZmP5CS3*. In contrast, nine SNPs in *ZmP5CS4* showed significant associations with enzyme activity (*p* < 0.01): chr8_167717555, chr8_167717571, chr8_167717835, chr8_167718085, chr8_167719263, chr8_167720316, chr8_167720431, chr8_167721389, and chr8_167721525.

Based on these nine SNPs, three haplotypes were defined (Table S1): HapA (14 lines), HapB (263 lines), and HapC (1 line). HapB was the predominant haplotype, with a population frequency of 94.6%, while HapA occurred at a frequency of 5.04%.

Phenotypic comparisons under salt stress revealed clear haplotype-based differences (Figure 7C,D). The mean relative P5CS enzyme activity in HapA carriers (11.03) was significantly higher than in HapB carriers (1.90) (*p* < 0.001). HapC was excluded from the statistical analysis as it only had one representative. Similarly, relative proline content under salt stress differed significantly between HapA and HapB (*p* < 0.001), and HapA was associated with lower standard evaluation scores (Figure S1). These results indicate that HapA is associated with elevated P5CS activity and enhanced proline accumulation under salt stress, potentially contributing to improved salt tolerance in maize.

### 2.9. Characterization of Salt Tolerance in Transgenic Maize

To elucidate the biological function of *ZmP5CS4* in salt stress adaptation, transgenic maize lines overexpressing this gene (ZmP5CS4-OE) and CRISPR-Cas9-mediated knockout lines (ZmP5CS4-KO) were generated and assessed alongside the wild type (WT) under control and salt stress conditions (200 mmol∙L^−1^ NaCl, 3 days).

**Figure 8:**
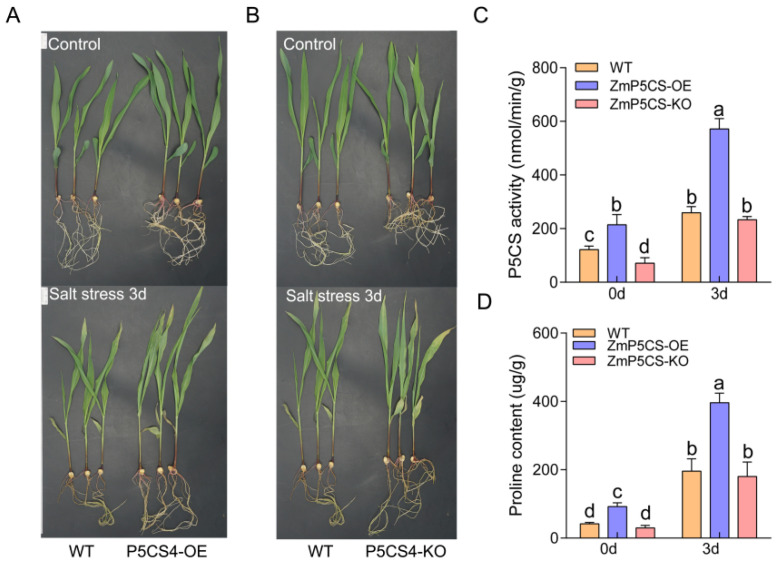
Functional analysis of *ZmP5CS4* in transgenic maize under salt stress. (**A**) Phenotypes of WT and ZmP5CS4-OE plants under control and salt stress conditions. (**B**) Phenotypes of WT and ZmP5CS4-KO plants under control and salt stress conditions. (**C**) P5CS enzyme activity in WT, ZmP5CS4-OE, and ZmP5CS4-KO plants. (**D**) Proline content in WT, ZmP5CS4-OE, and ZmP5CS4-KO plants. Different lower-case letters indicate statistically significant differences based on an ANOVA followed by Tukey’s HSD (*p* < 0.05).

Under non-stress conditions, no significant morphological or growth differences were observed among the WT, ZmP5CS4-OE, and ZmP5CS4-KO. However, after salt stress treatment, clear phenotypic differences emerged. ZmP5CS4-OE maintained higher leaf elongation rates and leaf greenness, as well as milder stress symptoms, than the WT (Figure 8A). In contrast, ZmP5CS4-KO exhibited severe growth inhibition, leaf curling, and premature senescence with pronounced yellowing, consistent with a salt-sensitive phenotype (Figure 8B).

Physiological and biochemical analyses under control conditions revealed significant genotypic differences in P5CS enzyme activity and Pro content among the WT, ZmP5CS4-OE, and ZmP5CS4-KO (Figure 8C,D). ZmP5CS4-OE showed the highest P5CS activity—1.76 times that of the WT and 3.01 times that of ZmP5CS4-KO. The Pro content in the WT was higher than that in ZmP5CS4-KO, but the difference was not significant; meanwhile, the Pro content in ZmP5CS4-OE was 1.22 times higher than in the WT and 2.11 times higher than in ZmP5CS4-KO.

Under salt stress, P5CS activity and proline content increased significantly in all lines relative to the control, with ZmP5CS4-OE maintaining higher levels than both the WT (1.21 and 1.45 times greater, respectively) and ZmP5CS4-KO (1.03 and 1.20 times greater, respectively). However, P5CS activity and proline content in the WT were higher than those in ZmP5CS4-KO, but the differences were not significant.

These results demonstrate that *ZmP5CS4* positively regulates P5CS activity and promotes proline biosynthesis under salt stress. Its overexpression enhances proline accumulation, improving osmotic adjustment and redox buffering capacity, thereby alleviating salt-induced damage. Under control conditions, loss of *ZmP5CS4* function reduces the efficiency of this pathway and decreases proline biosynthesis.

## 3. Discussion

### 3.1. Functional Divergence in Expression Patterns Among P5CS Gene Family Members in Maize

In plants such as Arabidopsis, Alfalfa, and sorghum, genes encoding *P5CS*—the key rate-limiting enzyme in proline biosynthesis—are generally represented by two or more highly homologous members (Table S2). These members are functionally distinct rather than redundant and display clear subfunctionalization. Typically, one member (*AtP5CS2* in Arabidopsis, *MtP5CS1* in alfalfa, *SbP5CS2* in sorghum) is constitutively expressed under non-stress conditions, maintaining basal proline levels and fulfilling its housekeeping role. In contrast, another member (*AtP5CS1* in Arabidopsis, *MtP5CS2/3* in alfalfa, *SbP5CS1* in sorghum) is strongly upregulated under stresses such as salinity, drought, or low temperature, thereby driving the substantial accumulation of stress-responsive proline [34,37,38].

**Figure 9:**
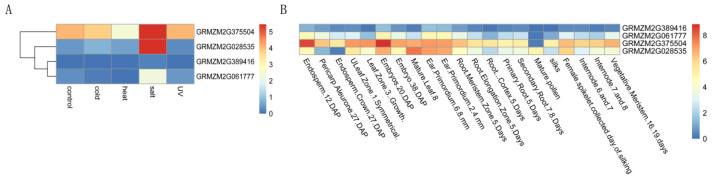
Gene expression analysis of *ZmP5CS* in different tissue locations under various stress conditions. (**A**) Heatmap of *ZmP5CS* expression in response to cold, heat, salt, and UV stress in seedlings of inbred lines B37. (**B**) Heatmap of *ZmP5CS* expression in different tissues.

Similar functional differentiation was observed in this study. Analysis of expression profiles in maize seedlings under salt, heat, cold, and UV stress (Figure 9A) revealed distinct patterns among *P5CS* members [36]: *ZmP5CS1* (Clade I, retaining only a few core motifs) and *ZmP5CS2* (Clade IV, lacking typical catalytic motifs and containing unique motifs) showed no significant expression changes across treatments, suggesting that motif variations or losses may be associated with functional specialization. In contrast, *ZmP5CS3* (Clade IV), which is predicted to be involved in steady-state synthesis within chloroplasts, maintained high constitutive expression under both control and stress conditions and was further upregulated under salt stress. *ZmP5CS4* (Clade III), potentially involved in membrane-associated rapid stress sensing and signaling, was significantly induced by salt stress. Based on their expression profiles, these evolutionarily divergent members appear to constitute a multi-layered, coordinated regulatory network for proline synthesis in maize. *ZmP5CS1* and *ZmP5CS2* likely function as constitutively expressed genes, and *ZmP5CS4* acts as a stress-responsive gene, while *P5CS3* may play a dual role in both basal synthesis and stress-induced accumulation. Subsequent qRT-PCR validation confirmed that *ZmP5CS1* and *ZmP5CS2* expression remained stable following stress, whereas *ZmP5CS4* was markedly upregulated, consistent with the predicted expression patterns. These differences are likely governed by distinct transcriptional regulatory mechanisms [39]. Furthermore, candidate gene association analysis supported this functional classification: *ZmP5CS1* and *ZmP5CS2* (constitutive genes), as well as *ZmP5CS3* (dual-function gene), did not show a significant correlation between natural sequence variation within their coding regions and relative enzyme activity under stress conditions.

Functional divergence within the *P5CS* gene family is further reflected in the diversity of spatiotemporal expression patterns across species. In Arabidopsis, for instance, *AtP5CS1* is widely expressed in most organs, whereas *AtP5CS2* is predominantly enriched in tissues with active cell division [34]. Under salt stress, *P5CS* expression in Phragmites Australis is upregulated in leaves but shows a downward trend in roots [40]. In sorghum responding to salt and drought stress, *SbP5CS1* and *SbP5CS2* display distinct spatiotemporal profiles: *SbP5CS2* is constitutively expressed, while *SbP5CS1* peaks in mature vegetative and reproductive organs [41]. Similarly, within the maize *P5CS* gene family, members exhibit pronounced expression differences [36] (Figure 9B). *ZmP5CS1* and *ZmP5CS2* maintain low expression throughout the growth cycle, potentially participating in basal physiological processes. *ZmP5CS3* shows exceptionally high expression in roots and vegetative meristems, suggesting a pivotal role in vegetative growth and grain development. In contrast, *ZmP5CS4* is highly expressed in mature leaves, likely supporting leaf-specific physiological functions. This pattern of differential expression across species, tissues, and stress conditions, together with non-redundant functions, represents a core strategy enabling plants to precisely and flexibly regulate proline metabolism in response to complex environments. However, such adaptive regulation may entail certain trade-offs with respect to crop yield formation.

### 3.2. The Dual Role of Proline in Maize Salt Stress Response and Its Implications for Breeding

Proline accumulation under abiotic stress conditions has been extensively documented across plant species, with concentrations reaching millimolar levels—representing several-fold increases compared to unstressed conditions [42]. A significant positive correlation has been widely reported between proline accumulation and plant tolerance to abiotic stress [19,21,43,44]. For instance, under drought stress, free-proline accumulation becomes particularly pronounced in wheat and fava bean, accounting for approximately 70% of total free amino acids. Drought-tolerant genotypes generally exhibit higher proline levels than susceptible ones, indicating a close association between proline accumulation and drought resistance [45]. Under salt stress, proline is often considered a potential physiological marker of salt tolerance, contributing to protective mechanisms such as osmotic adjustment and reactive-oxygen-species scavenging [46,47,48]. A comparable trend was observed in the present study (Figure S2): Prolonged exposure to salt stress resulted in increased *P5CS* enzyme activity, which was positively correlated with higher proline accumulation. However, greater proline accumulation in seedlings did not exhibit a statistically significant correlation with enhanced stress tolerance.

Nevertheless, recent studies indicate that the relationship between proline accumulation and salt tolerance is not invariably straightforward and positive. Multiple investigations have demonstrated that in certain species, salt-sensitive varieties accumulate higher proline under stress compared to salt-tolerant ones. For example, in switchgrass, proline concentration increases approximately 5000-fold in salt-sensitive lines, whereas salt-tolerant lines exhibit a comparatively modest increase [49]. Similar patterns have been reported in crops such as rice [50], barley [51], soybean [52], and cashew [53]. Furthermore, in salt-tolerant Paulownia, the highest proline accumulation was observed under low salinity (40 mM NaCl), while a significant decline occurred under high salinity [53]. These observations suggest that, under certain conditions, proline accumulation may reflect stress-induced injury rather than adaptive tolerance, and its physiological functions exhibit pronounced species- and genotype-dependent specificity [54].

An intriguing pattern emerged from the haplotype analysis of *ZmP5CS4.* The superior haplotype HapA, associated with significantly higher P5CS enzyme activity and proline accumulation under salt stress (Figure 7), was carried by only 14 out of 278 inbred lines (5.04%), while HapB predominated at 94.6%. This skewed distribution suggests that during long-term breeding selection, the high-activity HapA allele may have been inadvertently counter-selected in most elite germplasms. Notably, widely cultivated Chinese varieties such as Zhengdan958 and its parental lines Zheng58 and Chang7-2, as well as well-known inbred lines including Ji853 and Huangzao4, lack this superior haplotype. This observation raises an important question: why has a haplotype conferring enhanced stress-responsive proline accumulation not been fixed in elite breeding populations?

One plausible explanation lies in the potential trade-off between stress tolerance and agronomic performance, particularly yield. Proline biosynthesis is metabolically costly, consuming glutamate and NADPH—resources that are also essential for growth and grain filling. Under non-stress conditions, constitutively high P5CS activity may divert nitrogen and carbon skeletons away from primary metabolism, potentially compromising yield potential. This trade-off hypothesis is supported by observations in other species. In rice, *P5CS* overexpression did not demonstrate the expected utility for drought tolerance breeding, suggesting that excessive proline accumulation may impose fitness costs under non-stress conditions [55]. In Arabidopsis, although *P5CS1* overexpression enhanced stress tolerance, it also led to developmental abnormalities and reduced seed set under optimal conditions [35]. These findings indicate that while proline has a protective function under stress, its overaccumulation may disrupt metabolic homeostasis, particularly by altering the flux of glutamate and glutamine, with consequent adverse effects on crop yield and quality.

In maize, both *ZmP5CS3* and *ZmP5CS4* are expressed during the yield-forming stage. Under stress conditions, high *ZmP5CS4* expression may elevate glutamate consumption in source tissues, potentially affecting nitrogen remobilization to developing grains. This metabolic competition could explain the observed haplotype distribution: in environments where salt stress is not a primary constraint, genotypes carrying HapA may experience a yield penalty, leading to their gradual elimination from breeding programs focused on maximizing productivity. Conversely, under saline conditions, the stress-protective benefits of HapA may outweigh its metabolic costs, suggesting context-dependent value for this allele.

To test this hypothesis, future research should directly assess the relationship between *ZmP5CS4* allelic variation, proline dynamics, and yield components under field conditions. Field trials comparing near-isogenic lines carrying HapA versus HapB, across multiple environments with varying salinity levels, would clarify whether the superior haplotype confers yield advantages under stress without penalty under optimal conditions. Metabolomic analyses could further elucidate the impact of different alleles on primary carbon–nitrogen metabolism, identifying potential metabolic trade-offs associated with enhanced proline accumulation. Such studies would provide an empirical basis for deploying *ZmP5CS4* in breeding programs, potentially through allele-specific markers or gene-editing approaches that achieve stress-responsive expression without constitutive metabolic burden.

In conclusion, strategic fine-tuning of *P5CS* expression—whether through haplotype selection, promoter engineering, or coordinated regulation within the broader genetic network—offers a more effective and sustainable approach to improving salt tolerance than simply overexpressing a single gene. To this end, the present study establishes *ZmP5CS4* as a key target while highlighting the importance of understanding context-dependent trade-offs in stress adaptation.

## 4. Materials and Methods

### 4.1. Identification and Comprehensive Bioinformatics Analysis of the Maize P5CS Gene Family

Genomic data, including coding sequences, protein sequences, and annotation files for maize (*Zea mays* L.), rice (*Oryza sativa* L.), and sorghum (*Sorghum bicolor* L.), were retrieved from the Ensembl Plants database. Corresponding data for Arabidopsis thaliana were obtained from the TAIR10 database. For the systematic identification of *P5CS* gene family members in maize, a dual-screening strategy was employed. First, a local BLASTP search against the maize proteome was performed using TBtools 2.39 [56], with the amino acid sequences of two canonical Arabidopsis *P5CS* proteins as queries. To maximize coverage and minimize omissions, complementary hidden Markov model (HMM)-based screening was performed. After merging the results from both screening steps and removing duplicates, all candidate sequences were validated using online tools including NCBI CDD (Conserved Domains Database), Pfam, and SMART (SMART: Main page) to confirm the presence of functional P5CS domains. The identified genes were named sequentially according to their chromosomal positions.

The molecular weight (MW), theoretical isoelectric point (pI), and instability index (II) of the identified proteins were predicted using the ExPASy ProtParam tool (https://web.expasy.org/protparam/) [57]; subcellular localization was predicted using WoLF PSORT (https://wolfpsort.hgc.jp/) [58] and CELLO v.2.5 [59]; and the presence and cleavage sites of signal peptides were predicted using SignalP 5.0 [60]. Based on the maize GFF3 annotation file from Ensembl Plants, the chromosomal locations of the genes were determined using the “Gene Location Visualise from GFF” function in TBtools, and the resulting map was refined using Adobe Illustrator 2025.

To investigate evolutionary relationships, *P5CS* amino acid sequences were collected from maize, Arabidopsis, rice, and sorghum. Multiple sequence alignment was performed using MEGA 7 [61], and a phylogenetic tree was constructed using the Neighbor-Joining (NJ) method [62]. Phylogenetic analysis was conducted using the Poisson correction model, with pairwise deletion and a bootstrap test (1000 replicates). The final tree was visualized and optimized using the iTOL online platform.

Gene structure was analyzed using TBtools based on the GFF3 file. Conserved motifs were identified using the online tool MEME, and both the gene structures and motif distributions were integrated and visualized using TBtools. Promoter sequences for each gene were extracted using TBtools and submitted to the PlantCARE database for the prediction of cis-acting regulatory elements (CREs), with their distribution features visualized using the charting functions in TBtools. Syntenic relationships within the maize genome and homologous syntenic relationships between maize and Arabidopsis, rice, or sorghum were analyzed using the MCScanX algorithm built into TBtools, and the results were also visualized using TBtools. The secondary structure composition of the proteins was predicted using the SOPMA online server. Three-dimensional structure modeling was performed based on homologous templates using the Swiss-Model online platform. Finally, the protein sequences encoded by the four predicted homologous genes were submitted to the STRING database to construct a protein–protein Interaction (PPI) network, which was then visualized and edited using R 4.1.2 software.

### 4.2. Transcriptome Data Analysis and Stress Treatment

Using maize inbred line B73 as the material, plump and uniformly sized seeds were selected, surface-sterilized with a 0.1% HgCl solution for 10 min, and subsequently sown in a seedling cultivation system. The seedlings were cultivated under the following conditions: a 16 h light/8 h dark photoperiod, day/night temperatures of 25 ± 2 °C/20 ± 2 °C, relative humidity of approximately 60%, and a photosynthetic photon flux density of 50–60 μmol∙m^−2^∙s^−1^. During cultivation, the plants were regularly irrigated with half-strength Hoagland nutrient solution adjusted to pH 7.0. When the seedlings reached the three-leaf stage, salt stress treatment was initiated: the treatment group was irrigated with half-strength Hoagland nutrient solution containing 200 mmol∙L^−1^ NaCl (pH ≈ 6.8), while the control group continued to receive the original nutrient solution. Leaf samples (approximately 25 mg each) were collected at 0 h (pre-treatment), 1 day, and 3 days after treatment. After collection, samples were immediately flash-frozen in liquid nitrogen and stored at −80 °C, resulting in control and salt-stressed sample sets. Three biological replicates were established for each treatment.

### 4.3. RNA Extraction and Gene Expression Analysis

Total RNA was extracted from B73 leaves using the EZ-10 Spin Column Total RNA Miniprep Kit (Sangon Biotech, Shanghai, China), with three independent biological replicates established for each treatment. Subsequently, first-strand cDNA was synthesized from the extracted RNA using the MightyScript First-Strand cDNA Synthesis Master Mix (Sangon Biotech) and served as the template for qRT-PCR. The maize *Ubiquitin* (*ZmUbi*) gene was used as the internal reference. Primers were designed using Primer 5.0 software and synthesized by Sangon Biotech (Table S3), and qRT-PCR was performed using 2 × SGFast qPCR Master Mix (Low Rox) (Sangon Biotech) on an ABI 7500 Real-Time PCR System. The 20 μL reaction mixture contained 10 μL of 2 × SGFast qPCR Master Mix (Low Rox), 0.4 μL each of forward and reverse primers, 1 μL of cDNA template, and 8.2 μL of nuclease-free water. The thermal cycling protocol was as follows: initial denaturation at 95 °C for 3 min, followed by 40 cycles of denaturation at 95 °C for 3 s and combined annealing/extension at 60 °C for 30 s (with the fluorescence signal acquired during this step). The relative expression levels of the target genes were calculated using the 2^−ΔΔ^Ct method and are presented as the mean ± standard deviation.

### 4.4. Candidate Gene Association Analysis

The study employed 278 maize inbred lines as experimental materials, with seeds provided by the Maize Research Institute of the Jilin Academy of Agricultural Sciences. Seeds were selected for uniformity in size and fullness. After surface sterilization with 0.1% mercuric chloride for 10 min, the seeds were sown in a maize seedling phenotyping system (Chinese Patent No.: ZL200920177285.0) and grown in a light-controlled incubator. The growth conditions were as follows: a 16 h light/8 h dark photoperiod, day/night temperatures of (25 ± 2) °C/(20 ± 2) °C, relative humidity of 60%, and light intensity of 50–60 μmol∙m^−1^∙s^−1^. During cultivation, Hoagland nutrient solution was supplied regularly, and when seedlings reached the three-leaf stage, salt stress treatment groups and control groups were established. The stress group was irrigated with Hoagland solution containing 200 mmol∙L^−1^ NaCl, while the control group received standard Hoagland solution. The treatment solutions were refreshed every two days. Samples were collected at 0 h and 3 d after stress initiation to measure *P5CS* enzyme activity and proline content using commercial assay kits (P5CS Activity Assay Kit, Cat# G0819W; Proline Content Assay Kit, Cat# G0109W; Suzhou Greeth Biotech Co., Ltd., Suzhou, China). Seedling growth was observed and recorded daily for 7 consecutive days starting from the onset of salt stress (standard evaluation scores are provided in Table S4). Based on resequencing data from Wang et al. [63], we compiled allelic variation information for four *ZmP5CS* genes and their promoter regions (2 kb upstream) across the 278 maize inbred lines (Table S5). Association analysis between allelic variations in *ZmP5CS* genes and *P5CS* enzyme activity was performed using a mixed linear model (MLM) implemented in TASSEL5 software. This model accounted for potential false positives due to population structure (Q) and kinship (K). SNP chromosomal positions were mapped according to the B73 reference genome (B73 RefGen_V3).

### 4.5. Generation of Transgenic Maize via Overexpression

Based on the *P5CS4* gene sequence from the reference genome, the overexpression vector pCAM3300 Ubi P5CS was constructed. In this vector, the *ZmP5CS* gene is driven by the ZmUbi promoter, while the bar selectable marker is expressed under the control of the 35S promoter. The constructed plant expression vector was introduced into maize line B73 through Agrobacterium-mediated transformation. Transformed plants were screened via herbicide spraying, and positive individuals were self-pollinated to obtain the T_1_ transgenic generation. Genomic DNA was extracted from T_1_ plants and analyzed by PCR using primers specific to the target gene. Plants that tested positive for the target gene were again self-pollinated to produce the T_2_ generation for subsequent phenotypic evaluation. The methods employed for seedling cultivation and stress treatments were the same as those described in Section 2.4.

### 4.6. Generation of CRISPR-Cas9 Knockout Lines

Based on the *ZmP5CS4* gene sequence, two target sequences were designed: 5′-CCTACGCGAGGCTCCAGCCG-3′ and 5′-GCGTGGAGTGGATATCCGAA-3′. A gene-editing vector was constructed, in which Cas9 protein expression was driven by the *ZmUbi* promoter, and the expression of the selectable marker gene bar was driven by the 35S promoter. The constructed plant expression vector was introduced into maize line B73 through Agrobacterium-mediated transformation. To confirm the transformation, genomic DNA was extracted from the regenerated plants. Subsequently, PCR analysis was performed using gene-editing-detection primers, followed by agarose gel electrophoresis. It was observed that, compared to the wild type, the fragment size of the gene knockout materials was amplified to 332 bp, while that of the wild type was 674 bp. Genomic DNA was extracted from the T_0_ transformed materials, and PCR detection of the gene-editing sequence was carried out. Materials with gene-editing effects were selected and self-pollinated to obtain T_1_-generation edited materials. Genomic DNA was then extracted from the T_1_-generation materials, and PCR detection of both the gene-editing sequence and the transgene (CRISPR/Cas9) was performed. Individuals carrying homozygous gene-edited genotypes and lacking the transgene (CRISPR/Cas9) marker were selected and self-pollinated to obtain T_2_-generation gene-edited materials for subsequent phenotypic evaluation. The seedling culture method and stress treatment protocols remained consistent with those described in Section 2.4.

## 5. Conclusions

In this study, four members of the maize *ZmP5CS* gene family were systematically identified. Among them, *ZmP5CS4* was characterized as a key regulator of the salt stress response. This conclusion is supported by its strong stress-induced expression, the enrichment of stress-responsive cis-elements in its promoter region, and functional validation through transgenic approaches: *ZmP5CS4* overexpression enhanced salt tolerance, whereas its knockout increased salt sensitivity. Haplotype analysis of a natural population revealed an elite haplotype, HapA, which was defined by nine SNPs and present in only 5.04% of the inbred lines examined. This haplotype was significantly associated with higher P5CS enzyme activity and proline accumulation under salt stress. The low frequency of HapA in elite germplasm, including its absence in widely cultivated varieties such as Zhengdan958, suggests a potential trade-off between stress tolerance and agronomic performance under non-stress conditions. From a breeding perspective, the nine SNPs defining HapA constitute a practical molecular tool for marker-assisted selection to improve salt tolerance in maize. Future research investigating the interactions between *ZmP5CS4* and other stress-responsive genes—particularly those involved in osmotic regulation and hormone signaling—could further elucidate the regulatory network underlying salt adaptation and facilitate the development of gene stacking strategies for enhanced crop resilience. Collectively, this work advances our understanding of proline-mediated stress adaptation in maize and provides both a theoretical foundation and tangible genetic resources for breeding programs focused on improving salt tolerance.

## Figures and Tables

**Table 1:** Molecular characterization of *P5CS* gene families in Arabidopsis, maize, rice, and sorghum.

Gene ID	Chr.	Protein Length	Molecular Weight (kDa)	Isoelectric Point	Instability Index	GRAVY	Subcellular Localization	Aliphatic Index
*GRMZM2G389416*	2	362	39,224.30	9.52	58.41	−0.104	Cyto	99.67
*GRMZM2G061777*	6	117	12,542.03	5.54	30.66	−0.044	Cyto	84.19
*GRMZM2G375504*	8	717	77,708.14	6.11	30.26	−0.060	Chlo	101.31
*GRMZM2G028535*	8	731	78,794.41	5.95	29.51	−0.016	Plas	99.95
*Os01t0848200*	1	735	79,508.28	6.10	23.35	−0.058	Chlo	100.97
*Os05t0455500*	5	716	77,745.26	6.37	33.02	−0.060	Chlo	104.04
*SORBI_3003G356000*	3	729	78,361.73	5.99	26.52	−0.040	Plas	99.96
*SORBI_3009G160100*	9	716	77,743.20	5.99	33.13	−0.046	Chlo	104.33
*AT2G39800*	2	717	77,701.84	5.89	33.53	−0.072	Chlo	102.71
*AT3G55610*	3	726	78,871.35	6.35	33.85	−0.092	E.R.	101.27

**Table 2:** The secondary structure of *P5CS* proteins in maize.

Protein Name	Alpha Helix (%)	Extend Strand (%)	Beta Turn (%)	Random Coil (%)
GRMZM2G389416	35.08	17.40	7.46	40.06
GRMZM2G061777	35.90	20.51	5.13	38.46
GRMZM2G028535	49.66	18.88	6.16	25.31
GRMZM2G375504	48.68	18.55	5.86	26.92

## Data Availability

The original contributions presented in this study are included in the article. Further inquiries can be directed to the corresponding authors.

## Supplementary Materials

The following supporting information can be downloaded at https://www.mdpi.com/article/10.3390/plants15060946/s1. Figure S1: The standard evaluation scores (SESs) of HapA and HapB; Figure S2: Relationships among P5CS activity, proline content, and standard evaluation score; Table S1: The 278 maize inbred line haplotypes; Table S2: The number of P5CS gene family members in 14 crops, including *Zea mays* L.; Table S3: List of primers used in this study; Table S4: Modified standard evaluation score (SES) for salt tolerance at maize seedling stage; Table S5: Population structure of 278 inbred maize lines.
