# Supplementary material for: Identification of ZmP5CS Gene Family and Functional Analysis of ZmP5CS4 Under Salt Tolerance in Maize
Source: Plants (Basel). 2026 Mar 19;15(6):946. doi: 10.3390/plants15060946 (PMC13030422; doi:10.3390/plants15060946)
Supplement: Supplementary file 1 [file plants-15-00946-s001.zip › plants-4171420-supplementary.pdf]

Table S1. 278 Maize inbred line haplotypes

NO.	Inbred_name	Haplotype
1	Zheng58	TTCCGGGGTTGGCCGGGG
2	Chang7_2	TTCCGGGGTTGGCCGGGG
3	Qi319	TTCCGGGGTTGGCCGGGG
4	P138	TTCCGGGGTTGGCCGGGG
5	Ji853	TTCCGGGGTTGGCCGGGG
6	Lx9801	TTCCGGGGTTGGCCGGGG
7	Luyuan92	TTCCGGGGTTGGCCGGGG
8	515	TTCCGGGGTTGGCCGGGG
9	Dan340	TTCCGGGGTTGGCCGGGG
10	Ji533	TTCCGGGGTTGGCCGGGG
11	Mo17	TTCCGGGGTTGGCCGGGG
12	B73	TTCCGGGGTTGGCCGGGG
13	Oh43	TTCCGGGGTTGGCCGGGG
14	Xun92_8	TTCCGGGGTTGGCCGGGG
15	Ji876	TTCCGGGGTTGGCCGGGG
16	Ji63	TTCCGGGGTTGGCCGGGG
17	A188	TTCCGGGGTTGGCCGGGG
18	Ye52106	TTCCGGGGTTGGCCGGGG
19	Si273	TTCCGGGGTTGGCCGGGG
20	K55	TTCCGGGGTTGGCCGGGG
21	A801	TTCCGGGGTTGGCCGGGG
22	Baihunhuang	TTCCGGGGTTGGCCGGGG
23	Diangu11A	TTCCGGGGTTGGCCGGGG
24	Dong17	TTCCGGGGTTGGCCGGGG
25	Huangyesi3	TTCCGGGGTTGGCCGGGG
26	ZaC546	TTCCGGGGTTGGCCGGGG
27	Danhuang02	TTCCGGGGTTGGCCGGGG
28	K12	TTCCGGGGTTGGCCGGGG
29	K10	TTCCGGGGTTGGCCGGGG
30	Dong237	TTCCGGGGTTGGCCGGGG
31	Dian11	TTCCGGGGTTGGCCGGGG
32	E28	TTCCGGGGTTGGCCGGGG
33	Dan360	TTCCGGGGTTGGCCGGGG
34	Denghai4866	TTCCGGGGTTGGCCGGGG
35	4F1	TTCCGGGGTTGGCCGGGG
36	Lv28	TTCCGGGGTTGGCCGGGG
37	Dan9046	TTCCGGGGTTGGCCGGGG
38	7884_7Ht	TTCCGGGGTTGGCCGGGG
39	Longkang11	TTCCGGGGTTGGCCGGGG
40	Suwan1611	TTCCGGGGTTGGCCGGGG
41	Huobai	TTCCGGGGTTGGCCGGGG

42	Huangzaosi	TTCCGGGGTTGGCCGGGG
43	178	TTCCGGGGTTGGCCGGGG
44	478	TTCCGGGGTTGGCCGGGG
45	Ji842	TTCCGGGGTTGGCCGGGG
46	Tie7922	TTCCGGGGTTGGCCGGGG
47	Ye107	TTCCGGGGTTGGCCGGGG
48	Jing7	TTCCGGGGTTGGCCGGGG
49	5003	TTCCGGGGTTGGCCGGGG
50	Wu105	TTCCGGGGTTGGCCGGGG
51	Longkang1	TTCCGGGGTTGGCCGGGG
52	Yu82	TTCCGGGGTTGGCCGGGG
53	Zheng22	TTCCGGGGTTGGCCGGGG
54	Chang3	TTCCGGGGTTGGCCGGGG
55	HuangC	TTCCGGGGTTGGCCGGGG
56	8112	TTCCGGGGTTGGCCGGGG
57	C8605_2	TTCCGGGGTTGGCCGGGG
58	LD61	TTCCGGGGTTGGCCGGGG
59	Dan341	TTCCGGGGTTGGCCGGGG
60	Baihe43	TTCCGGGGTTGGCCGGGG
61	Liao2202	TTCCGGGGTTGGCCGGGG
62	Dan599	TTCCGGGGTTGGCCGGGG
63	Ye502	TTCCGGGGTTGGCCGGGG
64	Q1261	TTCCGGGGTTGGCCGGGG
65	Huang428	TTCCGGGGTTGGCCGGGG
66	OH40B	TTCCGGGGTTGGCCGGGG
67	8902	TTCCGGGGTTGGCCGGGG
68	Yan414	TTCCGGGGTTGGCCGGGG
69	Hai014	TTCCGGGGTTGGCCGGGG
70	B76	TTCCGGGGTTGGCCGGGG
71	Cheng18	TTCCGGGGTTGGCCGGGG
72	DH40	TTCCGGGGTTGGCCGGGG
73	Hua160	TTCCGGGGTTGGCCGGGG
74	Qi35	TTCCGGGGTTGGCCGGGG
75	Aijin525	TCCCGGGGTTGGCCGGGG
76	Liao9586	TTCCGGGGTTGGCCGGGG
77	A632	TTCCGGGGTTGGCCGGGG
78	B14	TTCCGGGGTTGGCCGGGG
79	B64	TTCCGGGGTTGGCCGGGG
80	H105W	TTCCGGGGTTGGCCGGGG
81	CM105	TTCCGGGGTTGGCCGGGG
82	B37	TTCCGGGGTTGGCCGGGG
83	32	TTCCGGGGTTGGCCGGGG
84	832	TTCCGGGGTTGGCCGGGG
85	Nan21_3	TTCCGGGGTTGGCCGGGG

86	488	TTCCGGGGTTGGCCGGGG
87	803	TTCCGGGGTTGGCCGGGG
88	5005	TTCCGGGGTTGGCCGGGG
89	Jian1495a	TTCCGGGGTTGGCCGGGG
90	Jian1495b	TTCCGGGGTTGGCCGGGG
91	9010	TTCCGGGGTTGGCCGGGG
92	446	TTCCGGGGTTGGCCGGGG
93	485	TTCCGGGGTTGGCCGGGG
94	812	TTCCGGGGTTGGCCGGGG
95	Hai1134	TTCCGGGGTTGGCCGGGG
96	Tai184	TTCCGGGGTTGGCCGGGG
97	Va26	TTCCGGGGTTGGCCGGGG
98	H95	TTCCGGGGTTGGCCGGGG
99	LH181	TTCCGGGGTTGGCCGGGG
100	LH213	TTCCGGGGTTGGCCGGGG
101	Lx03_2	TTCCGGGGTTGGCCGGGG
102	196	TTCCGGGGTTGGCCGGGG
103	Tian4	CCGGAAAAAAAATTAAAA
104	200B	TTCCGGGGTTGGCCGGGG
105	Baiye4	CCGGAAAAAAAATTAAAA
106	83IBI3	TTCCGGGGTTGGCCGGGG
107	PHJ90	TTCCGGGGTTGGCCGGGG
108	PHP55	TTCCGGGGTTGGCCGGGG
109	29MIBZ2	TTCCGGGGTTGGCCGGGG
110	PHJ65	TTCCGGGGTTGGCCGGGG
111	PH207	TTCCGGGGTTGGCCGGGG
112	PHV07	TTCCGGGGTTGGCCGGGG
113	911	TTCCGGGGTTGGCCGGGG
114	OQ403	TTCCGGGGTTGGCCGGGG
115	LIBC_4	TTCCGGGGTTGGCCGGGG
116	NQ508	TTCCGGGGTTGGCCGGGG
117	87_1	TTCCGGGGTTGGCCGGGG
118	Xinaiguang10	TTCCGGGGTTGGCCGGGG
119	Cheng687	TTCCGGGGTTGGCCGGGG
120	811A	TTCCGGGGTTGGCCGGGG
121	Shen137	TTCCGGGGTTGGCCGGGG
122	PHN82	TTCCGGGGTTGGCCGGGG
123	PHR58	TTCCGGGGTTGGCCGGGG
124	PHWG5	TTCCGGGGTTGGCCGGGG
125	PHBA6	TTCCGGGGTTGGCCGGGG
126	HZ85	TTCCGGGGTTGGCCGGGG
127	Ken44	TTCCGGGGTTGGCCGGGG
128	FusheA	TTCCGGGGTTGGCCGGGG
129	C14	CCGGAAAAAAAATTAAAA

130	48_2	CCGGAAAAAAAATTAAAA
131	Dahuang46	TTCCGGGGTTGGCCGGGG
132	Lv9kuan	TTCCGGGGTTGGCCGGGG
133	Yuanwu02	CCGGAAAAAAAATTAAAA
134	Aiji205	TTCCGGGGTTGGCCGGGG
135	Lv10	TTCCGGGGTTGGCCGGGG
136	Luosu3	TTCCGGGGTTGGCCGGGG
137	BZN	TTCCGGGGTTGGCCGGGG
138	CT52C	TTCCGGGGTTGGCCGGGG
139	Fu8501	TTCCGGGGTTGGCCGGGG
140	M9	TTCCGGGGTTGGCCGGGG
141	Y223	TTCCGGGGTTGGCCGGGG
142	27_263	TTCCGGGGTTGGCCGGGG
143	334_1	TTCCGGGGTTGGCCGGGG
144	Ernan24	TTCCGGGGTTGGCCGGGG
145	Ying64	TTCCGGGGTTGGCCGGGG
146	Zaodahuang	TTCCGGGGTTGGCCGGGG
147	434	TTCCGGGGTTGGCCGGGG
148	4112	TTCCGGGGTTGGCCGGGG
149	7327	TTCCGGGGTTGGCCGGGG
150	3H_2	TTCCGGGGTTGGCCGGGG
151	698_3	TTCCGGGGTTGGCCGGGG
152	75_1	CCGGAAAAAAAATTAAAA
153	H152	TTCCGGGGTTGGCCGGGG
154	Shuang741	TTCCGGGGTTGGCCGGGG
155	Ye8001	TTCCGGGGTTGGCCGGGG
156	Yuanqi123	CCGGAAAAAAAATTAAAA
157	Chang72	TTCCGGGGTTGGCCGGGG
158	618	TTCCGGGGTTGGCCGGGG
159	9058	TTCCGGGGTTGGCCGGGG
160	Ay420	TTCCGGGGTTGGCCGGGG
161	DH65232	TTCCGGGGTTGGCCGGGG
162	912	TTCCGGGGTTGGCCGGGG
163	3IBZ2	TTCCGGGGTTGGCCGGGG
164	BCC03	TTCCGGGGTTGGCCGGGG
165	CS608	TTCCGGGGTTGGCCGGGG
166	E8501	TTCCGGGGTTGGCCGGGG
167	F42	TTCCGGGGTTGGCCGGGG
168	ICI_740	TTCCGGGGTTGGCCGGGG
169	LH128	TTCCGGGGTTGGCCGGGG
170	LH160	TTCCGGGGTTGGCCGGGG
171	LH191	TTCCGGGGTTGGCCGGGG
172	LH193	TTCCGGGGTTGGCCGGGG
173	LH194	TTCCGGGGTTGGCCGGGG

174	LH195	TTCCGGGGTTGGCCGGGG
175	LH202	TTCCGGGGTTGGCCGGGG
176	LH205	TTCCGGGGTTGGCCGGGG
177	LH212Ht	TTCCGGGGTTGGCCGGGG
178	LH214	TTCCGGGGTTGGCCGGGG
179	LH215	TTCCGGGGTTGGCCGGGG
180	LH220Ht	TTCCGGGGTTGGCCGGGG
181	Lp215D	TTCCGGGGTTGGCCGGGG
182	MBUB	TTCCGGGGTTGGCCGGGG
183	ML606	TTCCGGGGTTGGCCGGGG
184	MQ305	TTCCGGGGTTGGCCGGGG
185	OS602	TTCCGGGGTTGGCCGGGG
186	PHP76	TTCCGGGGTTGGCCGGGG
187	PHP85	TTCCGGGGTTGGCCGGGG
188	A554	TTCCGGGGTTGGCCGGGG
189	B95	TTCCGGGGTTGGCCGGGG
190	CM37	TTCCGGGGTTGGCCGGGG
191	CM7	TTCCGGGGTTGGCCGGGG
192	Mo24W	TTCCGGGGTTGGCCGGGG
193	Mt42	TTCCGGGGTTGGCCGGGG
194	ND255	TTCCGGGGTTGGCCGGGG
195	R2	TTCCGGGGTTGGCCGGGG
196	W9	TTCCGGGGTTGGCCGGGG
197	A71	CCGGAAAAAAAATTAAAA
198	KY228	TTCCGGGGTTGGCCGGGG
199	B57	TTCCGGGGTTGGCCGGGG
200	K4	TTCCGGAAAAAAATTAAAA
201	W23	CCGGAAAAAAAATTAAAA
202	K64	TTCCGGGGTTGGCCGGGG
203	PHH93	TTCCGGGGTTGGCCGGGG
204	A334	TTCCGGGGTTGGCCGGGG
205	C46	TTCCGGGGTTGGCCGGGG
206	A374	TTCCGGGGTTGGCCGGGG
207	R4	TTCCGGGGTTGGCCGGGG
208	PHGG7	TTCCGGGGTTGGCCGGGG
209	H49	TTCCGGGGTTGGCCGGGG
210	A171	CCGGAAAAAAAATTAAAA
211	MS153	TTCCGGGGTTGGCCGGGG
212	N6	CCGGAAAAAAAATTAAAA
213	B8	TTCCGGGGTTGGCCGGGG
214	H5	TTCCGGGGTTGGCCGGGG
215	C102	CCGGAAAAAAAATTAAAA
216	A375	TTCCGGGGTTGGCCGGGG
217	WF9	TTCCGGGGTTGGCCGGGG

218	R168	CCGGAAAAAAAATTAAAA
219	A659	TTCCGGGGTTGGCCGGGG
220	B98	TTCCGGGGTTGGCCGGGG
221	38_11	TTCCGGGGTTGGCCGGGG
222	Oh7	TTCCGGGGTTGGCCGGGG
223	W22	TTCCGGGGTTGGCCGGGG
224	OH7B	TTCCGGGGTTGGCCGGGG
225	B96	TTCCGGGGTTGGCCGGGG
226	ZS01250	TTCCGGGGTTGGCCGGGG
227	B12	CCGGAAAAAAAATTAAAA
228	OQ101	TTCCGGGGTTGGCCGGGG
229	LH190	TTCCGGGGTTGGCCGGGG
230	904	TTCCGGGGTTGGCCGGGG
231	LH162	TTCCGGGGTTGGCCGGGG
232	I_205	TTCCGGGGTTGGCCGGGG
233	B10	TTCCGGGGTTGGCCGGGG
234	M14	TTCCGGGGTTGGCCGGGG
235	2MA22	TTCCGGGGTTGGCCGGGG
236	RS_710	TTCCGGGGTTGGCCGGGG
237	PHW30	TTCCGGGGTTGGCCGGGG
238	H99	TTCCGGGGTTGGCCGGGG
239	6M502A	TTCCGGGGTTGGCCGGGG
240	R177	TTCCGGGGTTGGCCGGGG
241	N28	TTCCGGGGTTGGCCGGGG
242	PHM81	TTCCGGGGTTGGCCGGGG
243	B100	TTCCGGGGTTGGCCGGGG
244	F118	TTCCGGGGTTGGCCGGGG
245	CI_187_2	TTCCGGGGTTGGCCGGGG
246	LH163	TTCCGGGGTTGGCCGGGG
247	H84	TTCCGGGGTTGGCCGGGG
248	C123	TTCCGGGGTTGGCCGGGG
249	PHR55	TTCCGGGGTTGGCCGGGG
250	PHR30	TTCCGGGGTTGGCCGGGG
251	FBLA	TTCCGGGGTTGGCCGGGG
252	PHV53	TTCCGGGGTTGGCCGGGG
253	PHW43	TTCCGGGGTTGGCCGGGG
254	2FACC	TTCCGGGGTTGGCCGGGG
255	PHVA9	TTCCGGGGTTGGCCGGGG
256	PHJ40	TTCCGGGGTTGGCCGGGG
257	6F629	TTCCGGGGTTGGCCGGGG
258	CS405	TTCCGGGGTTGGCCGGGG
259	PHN66	TTCCGGGGTTGGCCGGGG
260	LH192	TTCCGGGGTTGGCCGGGG
261	PHJ89	TTCCGGGGTTGGCCGGGG

262	LH222	TTCCGGGGTTGGCCGGGG
263	PHPR5	TTCCGGGGTTGGCCGGGG
264	PHG35	TTCCGGGGTTGGCCGGGG
265	ICI_893	TTCCGGGGTTGGCCGGGG
266	LH209	TTCCGGGGTTGGCCGGGG
267	787	TTCCGGGGTTGGCCGGGG
268	NS501	TTCCGGGGTTGGCCGGGG
269	ICI_193	TTCCGGGGTTGGCCGGGG
270	LH208	TTCCGGGGTTGGCCGGGG
271	NL001	TTCCGGGGTTGGCCGGGG
272	PHT55	TTCCGGGGTTGGCCGGGG
273	IBB14	TTCCGGGGTTGGCCGGGG
274	LH196	TTCCGGGGTTGGCCGGGG
275	PHW51	TTCCGGGGTTGGCCGGGG
276	ICI_441	TTCCGGGGTTGGCCGGGG
277	LH206	TTCCGGGGTTGGCCGGGG
278	CI31A	TTCCGGGGTTGGCCGGGG

Table S2. number of *P5CS* gene family in 14 crops including *Zea mays* L.

Crop name	*Zea mays* L.	*Arabidopsis thaliana* L.	*Oryza sativa* L.	*Sorghum bicolor* L.	*Setaria italica* L.	*Hordeum vulgare* L.
Total	4	2	2	2	2	2
Crop name	*Triticum aestivum* L.	*Medicago truncatula*	*Aegilops tauschii*	*Brachypo diumdistachyon*	*Puccinellia tenuiflora*	*Chenopodium quinoa Willd.*
Total	6	3	1	2	2	1

Table S3 List of primers used in this study.

Primers for quantitative real-time PCR	
ZmUBI_F	TGGTTGTGGCTTCGTTGGTT
ZmUBI_R	GCTGCAGAAGAGTTTTGGGTACA
ZmP5CS1-RT-F	CGCAAGGAGCAAGACAG
ZmP5CS1-RT-R	AACACGGATAGTAGGTAGGC
ZmP5CS2-RT-F	GTTGGCATAAGCACAGG
ZmP5CS2-RT-R	CAGTTCACCGTCCCTCA
ZmP5CS3-RT-F	ACGAGGTGATCCTTGTGA
ZmP5CS3-RT-R	CATCCAGCTCCATCTGTG
ZmP5CS4-RT-F	CCAAGCGATCCTCAGTCA
ZmP5CS4-RT-R	TGCCACCTCTTCCAACAC
Primers for transgenic overexpression plants	
ZmP5CS3-OE-F	CAAGGAGCAAGACAGTCGTG
ZmP5CS3-OE-R	ACCATTGCCACTTCGAATCG

Primers for transgenic knockout plants

ZmP5CS3-crispr-F	CGTTGGTTGCTAGGATGACC
ZmP5CS3-crispr-R	GATTACCGCTTTCATCCCTACA

Table S4. Modified standard evaluation score (SES) for salt tolerance at maize seedlings stage

Score	Tolerance	Observation
1	Highly tolerant	Normal growth on leaf symptoms
2	Tolerant	Nearly normal growth, but leaf tips orfew leaves whitish and rolled
3	Moderately tolerant	Growth severely retarded; most leavesrolled; only a few are elongating
4	Sensitive	Complete cessation of growth; mostleaves dry; some plants dying
5	Highly sensitive	Almost all plants dead or dying

Table S5. Population Structure of 278 Maize Inbred Lines

NO.	Inbred_name	SS1	SS2	PA	X-group	Lancaster1	Lancaster2	TSPT	IDT	PB	Mixed
1	Zheng58	0.00001	0.00001	0.99991	0.00001	0.00001	0.00001	0.00001	0.00001	0.00001	0.00001
2	Chang7_2	0.00001	0.00001	0.00001	0.00001	0.00001	0.00001	0.99991	0.00001	0.00001	0.00001
3	Qi319	0.00001	0.000013	0.00001	0.00001	0.00001	0.00001	0.00001	0.00001	0.999907	0.00001
4	P138	0.00001	0.00001	0.00001	0.00001	0.00001	0.00001	0.00001	0.00001	0.99991	0.00001
5	Ji853	0.00001	0.00001	0.00001	0.00001	0.99991	0.00001	0.00001	0.00001	0.00001	0.00001
6	Lx9801	0.00001	0.00001	0.00001	0.00001	0.00001	0.012667	0.987253	0.00001	0.00001	0.00001
7	Luyuan92	0.387236	0.00001	0.00001	0.008579	0.548979	0.055147	0.00001	0.00001	0.00001	0.00001
8	515	0.015599	0.118866	0.00001	0.00001	0.00001	0.193603	0.40916	0.00001	0.006467	0.256267
9	Dan340	0.00001	0.00001	0.00001	0.00001	0.00001	0.99991	0.00001	0.00001	0.00001	0.00001
10	Ji533	0.99991	0.00001	0.00001	0.00001	0.00001	0.00001	0.00001	0.00001	0.00001	0.00001
11	Mo17	0.00001	0.00001	0.00001	0.00001	0.99991	0.00001	0.00001	0.00001	0.00001	0.00001
12	B73	0.99991	0.00001	0.00001	0.00001	0.00001	0.00001	0.00001	0.00001	0.00001	0.00001
13	Oh43	0.00001	0.00001	0.00001	0.000011	0.00001	0.999909	0.00001	0.00001	0.00001	0.00001
14	Xun92_8	0.00001	0.00001	0.00001	0.00001	0.00001	0.00001	0.99991	0.00001	0.00001	0.00001
15	Ji876	0.00001	0.540636	0.00001	0.00001	0.00001	0.459284	0.00001	0.00001	0.00001	0.00001
16	Ji63	0.043312	0.069409	0.00001	0.00001	0.048293	0.00001	0.017488	0.02318	0.00001	0.798279
17	A188	0.00001	0.056494	0.045037	0.048964	0.103062	0.050161	0.0468	0.062548	0.00001	0.586915
18	Ye52106	0.061433	0.053896	0.00001	0.015808	0.173006	0.087757	0.128889	0.00001	0.00001	0.479181
19	Si273	0.052671	0.00001	0.04878	0.00001	0.044998	0.523575	0.203817	0.00001	0.00001	0.126119
20	K55	0.00001	0.042022	0.063125	0.062819	0.03378	0.061922	0.019219	0.026439	0.00001	0.690655
21	A801	0.175674	0.00001	0.657447	0.00001	0.00001	0.00001	0.00001	0.00001	0.00001	0.166809
22	Baihunhuang	0.000279	0.00001	0.040722	0.153834	0.026503	0.019712	0.063018	0.00001	0.00001	0.695902
23	Diangu11A	0.04031	0.045486	0.00001	0.063605	0.210568	0.00001	0.010061	0.013652	0.00001	0.616287
24	Dong17	0.00001	0.00001	0.00001	0.00001	0.002006	0.00001	0.357973	0.046323	0.02137	0.572278
25	Huangyesi3	0.00001	0.00001	0.00001	0.00001	0.00001	0.00001	0.99991	0.00001	0.00001	0.00001
26	ZaC546	0.038797	0.00001	0.00001	0.199177	0.665555	0.00001	0.00001	0.00001	0.00001	0.096412
27	Danhuang02	0.02853	0.094082	0.15007	0.00001	0.00001	0.173097	0.022373	0.017035	0.00001	0.514783
28	K12	0.00001	0.137795	0.00001	0.00001	0.229654	0.00001	0.581859	0.00001	0.00001	0.050631
29	K10	0.00493	0.00001	0.020219	0.090218	0.00001	0.207546	0.04013	0.00001	0.00001	0.636917

30	Dong237	0.016705	0.046059	0.021057	0.175117	0.308607	0.00001	0.044542	0.000172	0.017838	0.369893
31	Dian11	0.00001	0.024234	0.00369	0.151414	0.143612	0.005863	0.00001	0.00001	0.00001	0.671147
32	E28	0.057629	0.111845	0.00001	0.00001	0.014932	0.131341	0.00001	0.00001	0.035726	0.648488
33	Dan360	0.00001	0.00001	0.008563	0.00001	0.048151	0.943216	0.00001	0.00001	0.00001	0.00001
34	Denghai4866	0.010906	0.00001	0.69378	0.00001	0.007719	0.00001	0.018738	0.007954	0.00001	0.260864
35	4F1	0.00001	0.00001	0.00001	0.00001	0.72549	0.155026	0.00001	0.01584	0.049792	0.053801
36	Lv28	0.006105	0.041386	0.00001	0.00001	0.00001	0.08008	0.035636	0.061406	0.00001	0.775348
37	Dan9046	0.00001	0.00001	0.00178	0.179219	0.085783	0.413424	0.00001	0.00001	0.319744	0.00001
38	7884_7Ht	0.051698	0.00001	0.00001	0.00001	0.017394	0.00001	0.233228	0.00001	0.697619	0.00001
39	Longkang11	0.00001	0.00001	0.00001	0.00001	0.501578	0.498342	0.00001	0.00001	0.00001	0.00001
40	Suwan1611	0.049331	0.019351	0.016953	0.00001	0.00001	0.164812	0.066107	0.002629	0.123071	0.557726
41	Huobai	0.000164	0.017053	0.004422	0.00001	0.027061	0.02332	0.180502	0.006357	0.034945	0.706164
42	Huangzaosi	0.000015	0.00001	0.00001	0.00001	0.00001	0.00001	0.999904	0.000011	0.00001	0.00001
43	178	0.00001	0.00001	0.00001	0.00001	0.00001	0.00001	0.00001	0.00001	0.99991	0.00001
44	478	0.00001	0.00001	0.999894	0.000015	0.00001	0.00001	0.000013	0.00001	0.00001	0.000017
45	Ji842	0.130575	0.005464	0.158627	0.198706	0.00001	0.060263	0.034867	0.00001	0.179462	0.232016
46	Tie7922	0.429854	0.12102	0.338535	0.00001	0.110524	0.000011	0.000016	0.00001	0.00001	0.00001
47	Ye107	0.08227	0.093183	0.020519	0.00001	0.279788	0.013136	0.00001	0.00001	0.049413	0.461661
48	Jing7	0.020039	0.034301	0.00001	0.00001	0.00001	0.00001	0.647146	0.012248	0.023263	0.262963
49	5003	0.00001	0.00001	0.751349	0.00001	0.00001	0.00001	0.00001	0.00064	0.00001	0.247941
50	Wu105	0.011878	0.00001	0.002671	0.00001	0.021015	0.00001	0.135624	0.026335	0.00001	0.802437
51	Longkang1	0.067798	0.00001	0.00001	0.004118	0.447075	0.184233	0.042755	0.033317	0.00001	0.220675
52	Yu82	0.00001	0.00001	0.678251	0.00001	0.00001	0.000015	0.321664	0.00001	0.00001	0.00001
53	Zheng22	0.101265	0.005005	0.036865	0.044112	0.00001	0.266132	0.408026	0.029112	0.059832	0.049642
54	Chang3	0.047881	0.00001	0.00001	0.075197	0.208953	0.00001	0.00001	0.00001	0.00001	0.66791
55	HuangC	0.00001	0.014168	0.00001	0.21813	0.263553	0.215553	0.00001	0.103301	0.032377	0.152887
56	8112	0.615482	0.00001	0.384426	0.00001	0.00001	0.00001	0.000017	0.000015	0.00001	0.00001
57	C8605_2	0.118319	0.250848	0.00001	0.035461	0.076576	0.315569	0.000011	0.00001	0.043946	0.15925
58	LD61	0.094681	0.00001	0.021137	0.00001	0.030388	0.782698	0.00001	0.00001	0.00001	0.071046
59	Dan341	0.020998	0.005602	0.253006	0.013679	0.015229	0.207202	0.00001	0.020006	0.163464	0.300803
60	Baihe43	0.009702	0.00001	0.059076	0.018472	0.016747	0.00001	0.14826	0.010577	0.020839	0.716308
61	Liao2202	0.00004	0.080089	0.250233	0.095661	0.00001	0.011287	0.00001	0.00001	0.297822	0.264838
62	Dan599	0.00001	0.00001	0.00001	0.00001	0.00001	0.00001	0.00001	0.00001	0.99991	0.00001
63	Ye502	0.00001	0.00001	0.00001	0.00001	0.00001	0.131368	0.868552	0.00001	0.00001	0.00001
64	Q1261	0.007587	0.156467	0.00001	0.00001	0.010808	0.00001	0.590112	0.00001	0.00001	0.234976
65	Huang428	0.030664	0.00001	0.00001	0.00001	0.00001	0.001812	0.740665	0.00001	0.011088	0.215721
66	OH40B	0.029649	0.011566	0.00001	0.077712	0.07995	0.629478	0.00001	0.00001	0.014023	0.157591
67	8902	0.249018	0.00001	0.373998	0.00001	0.193301	0.00001	0.058154	0.00001	0.062315	0.063173
68	Yan414	0.00001	0.00001	0.032752	0.042209	0.00001	0.117487	0.064135	0.00001	0.00001	0.743367
69	Hai014	0.00001	0.007697	0.00001	0.006531	0.00001	0.003871	0.174174	0.006442	0.023056	0.778199
70	B76	0.158827	0.377297	0.00001	0.033108	0.00001	0.061294	0.00001	0.006742	0.201108	0.161595
71	Cheng18	0.012432	0.00277	0.00001	0.00001	0.386265	0.129295	0.011341	0.00001	0.00001	0.457857
72	DH40	0.075144	0.001824	0.058514	0.030032	0.00001	0.288598	0.247675	0.002362	0.00001	0.295832
73	Hua160	0.050851	0.006376	0.011314	0.037061	0.00001	0.032673	0.060211	0.025098	0.00001	0.776395

74	Qi35	0.00001	0.00001	0.00001	0.00001	0.698213	0.00001	0.021799	0.002921	0.00001	0.277008
75	Aijin525	0.048918	0.00001	0.00001	0.00001	0.139396	0.005468	0.147624	0.00001	0.021545	0.637009
76	Liao9586	0.02622	0.00001	0.6133	0.00001	0.010074	0.350346	0.00001	0.00001	0.00001	0.00001
77	A632	0.00001	0.99991	0.00001	0.00001	0.00001	0.00001	0.00001	0.00001	0.00001	0.00001
78	B14	0.00001	0.99991	0.00001	0.00001	0.00001	0.00001	0.00001	0.00001	0.00001	0.00001
79	B64	0.00001	0.99991	0.00001	0.00001	0.00001	0.00001	0.00001	0.00001	0.00001	0.00001
80	H105W	0.00001	0.851705	0.00001	0.00001	0.00001	0.00001	0.00001	0.00001	0.00001	0.148215
81	CM105	0.00001	0.821618	0.00001	0.00001	0.00001	0.009948	0.00001	0.054623	0.00001	0.113751
82	B37	0.152942	0.486748	0.00001	0.036812	0.00001	0.017552	0.00001	0.00001	0.253267	0.052638
83	32	0.68587	0.00001	0.210676	0.00001	0.103383	0.00001	0.000012	0.00001	0.00001	0.00001
84	832	0.654559	0.00001	0.283644	0.00001	0.061727	0.00001	0.00001	0.00001	0.00001	0.00001
85	Nan21_3	0.131812	0.468339	0.00001	0.004829	0.026971	0.00001	0.00001	0.00001	0.00001	0.367998
86	488	0.00001	0.00001	0.99991	0.00001	0.00001	0.00001	0.00001	0.00001	0.00001	0.00001
87	803	0.174488	0.00001	0.825425	0.00001	0.00001	0.00001	0.00001	0.00001	0.000017	0.00001
88	5005	0.00001	0.00001	0.712102	0.002704	0.005014	0.00001	0.00001	0.01126	0.00001	0.268871
89	Jian1495a	0.00001	0.00001	0.647473	0.00001	0.00001	0.00001	0.00001	0.00001	0.204061	0.148396
90	Jian1495b	0.00001	0.00001	0.638084	0.011196	0.00001	0.00001	0.00001	0.00001	0.212804	0.137857
91	9010	0.00001	0.00001	0.072073	0.00001	0.042302	0.630384	0.00001	0.00001	0.00001	0.255182
92	446	0.000011	0.00001	0.00001	0.00001	0.00001	0.999909	0.00001	0.00001	0.00001	0.00001
93	485	0.000012	0.000017	0.00001	0.00001	0.999897	0.00001	0.00001	0.00001	0.00001	0.000013
94	812	0.560863	0.00001	0.405419	0.00001	0.00001	0.00001	0.033648	0.00001	0.00001	0.00001
95	Hai1134	0.242662	0.00001	0.051878	0.00001	0.33833	0.262738	0.00001	0.000143	0.104208	0.00001
96	Tai184	0.087888	0.059554	0.00001	0.00001	0.479202	0.278215	0.042716	0.00001	0.030487	0.021909
97	Va26	0.045433	0.000012	0.00001	0.004712	0.019345	0.750716	0.000011	0.000014	0.00001	0.179738
98	H95	0.007057	0.000018	0.00001	0.096065	0.00001	0.644622	0.00001	0.00001	0.007096	0.245101
99	LH181	0.00001	0.00001	0.00001	0.00001	0.920149	0.079771	0.00001	0.00001	0.00001	0.00001
100	LH213	0.00001	0.00001	0.00001	0.00001	0.867475	0.132445	0.00001	0.00001	0.00001	0.00001
101	Lx03_2	0.00001	0.00001	0.000017	0.00001	0.00001	0.00001	0.999903	0.000011	0.00001	0.00001
102	196	0.00001	0.000011	0.00001	0.000013	0.00001	0.180994	0.818917	0.000015	0.00001	0.00001
103	Tian4	0.000013	0.00001	0.00001	0.00001	0.006235	0.00001	0.787611	0.00001	0.00001	0.20608
104	200B	0.00001	0.040808	0.00001	0.00001	0.00001	0.00001	0.720332	0.00001	0.00001	0.23879
105	Baiye4	0.00001	0.00001	0.038582	0.00001	0.085698	0.00001	0.697075	0.00001	0.00001	0.178585
106	83IBI3	0.00001	0.00001	0.00001	0.00001	0.00001	0.00001	0.00001	0.99991	0.00001	0.00001
107	PHJ90	0.00001	0.00001	0.00001	0.00001	0.00001	0.00001	0.00001	0.99991	0.00001	0.00001
108	PHP55	0.00001	0.00001	0.00001	0.00001	0.00001	0.00001	0.00001	0.99991	0.00001	0.00001
109	29MIBZ2	0.00001	0.00001	0.00001	0.000012	0.00001	0.00001	0.00001	0.999908	0.00001	0.00001
110	PHJ65	0.00001	0.00001	0.00001	0.00001	0.00001	0.00001	0.00001	0.999908	0.00001	0.000012
111	PH207	0.00001	0.000012	0.00001	0.00001	0.00001	0.00001	0.000016	0.999902	0.00001	0.00001
112	PHV07	0.00001	0.00001	0.00001	0.131872	0.00001	0.00001	0.00001	0.868048	0.00001	0.00001
113	911	0.00001	0.00001	0.00001	0.045732	0.101415	0.040212	0.00001	0.812581	0.00001	0.00001
114	OQ403	0.00001	0.165272	0.00001	0.00001	0.00001	0.00001	0.00001	0.80842	0.026238	0.00001
115	LIBC_4	0.00001	0.00001	0.00001	0.160033	0.00001	0.064675	0.00001	0.775222	0.00001	0.00001
116	NQ508	0.041819	0.140706	0.00001	0.00001	0.00001	0.00001	0.00001	0.738549	0.078866	0.00001
117	87_1	0.00001	0.00001	0.00001	0.00001	0.00001	0.00001	0.00001	0.00001	0.99991	0.00001

118	Xinaiguang10	0.00001	0.00001	0.00001	0.00001	0.00001	0.00001	0.00001	0.00001	0.99991	0.00001
119	Cheng687	0.00001	0.00001	0.00001	0.00001	0.00001	0.00001	0.00001	0.00001	0.99991	0.00001
120	811A	0.00001	0.00001	0.000012	0.00001	0.00001	0.00001	0.00001	0.00001	0.999908	0.00001
121	Shen137	0.00001	0.000011	0.00001	0.00001	0.00001	0.00001	0.00001	0.00001	0.999908	0.000011
122	PHN82	0.00001	0.737877	0.00001	0.00001	0.102373	0.00001	0.00001	0.00001	0.15968	0.00001
123	PHR58	0.00001	0.00001	0.00001	0.99991	0.00001	0.00001	0.00001	0.00001	0.00001	0.00001
124	PHWG5	0.00001	0.00001	0.00001	0.99991	0.00001	0.00001	0.00001	0.00001	0.00001	0.00001
125	PHBA6	0.00001	0.00001	0.00001	0.676932	0.161319	0.161678	0.00001	0.00001	0.00001	0.00001
126	HZ85	0.00001	0.357397	0.00001	0.642523	0.00001	0.00001	0.00001	0.00001	0.00001	0.00001
127	Ken44	0.00001	0.049636	0.00001	0.049085	0.060392	0.00001	0.00001	0.065576	0.00001	0.77526
128	FusheA	0.00001	0.00001	0.00001	0.010025	0.00001	0.00001	0.246835	0.00001	0.041928	0.701152
129	C14	0.00001	0.030723	0.00001	0.010171	0.085075	0.036976	0.020626	0.129369	0.00001	0.687029
130	48_2	0.054963	0.00001	0.00001	0.00001	0.00001	0.044727	0.162196	0.00001	0.050758	0.687307
131	Dahuang46	0.004991	0.00001	0.00001	0.003992	0.011732	0.022086	0.017861	0.022532	0.00001	0.916775
132	Lv9kuan	0.07777	0.00001	0.00036	0.00001	0.00001	0.105467	0.019102	0.00001	0.002881	0.79438
133	Yuanwu02	0.00001	0.00001	0.00001	0.00001	0.008781	0.00001	0.222842	0.00001	0.00001	0.768307
134	Aiji205	0.030971	0.055496	0.055018	0.00001	0.034199	0.002058	0.036887	0.016552	0.052627	0.716183
135	Lv10	0.066975	0.008783	0.000597	0.055578	0.030635	0.056001	0.026662	0.047099	0.000365	0.707304
136	Luosu3	0.019702	0.085769	0.045173	0.049744	0.010253	0.00001	0.090368	0.004296	0.00001	0.694675
137	BZN	0.033247	0.071215	0.00001	0.103856	0.040866	0.091831	0.036167	0.012264	0.00001	0.610534
138	CT52C	0.00001	0.00001	0.00001	0.00001	0.40565	0.030021	0.00001	0.00001	0.221359	0.34291
139	Fu8501	0.056724	0.062406	0.013782	0.00001	0.496182	0.080542	0.00001	0.00001	0.027374	0.262961
140	M9	0.086828	0.204024	0.00001	0.055711	0.155005	0.28874	0.00001	0.00001	0.032823	0.176839
141	Y223	0.00001	0.00001	0.00001	0.358666	0.126769	0.00001	0.091848	0.00001	0.108753	0.313913
142	27_263	0.025374	0.00001	0.00001	0.00001	0.500277	0.00001	0.066319	0.091454	0.132381	0.184155
143	334_1	0.042937	0.000015	0.00001	0.021255	0.00001	0.56862	0.068952	0.004016	0.00001	0.294174
144	Ernan24	0.11446	0.00001	0.026442	0.00001	0.159225	0.005435	0.086242	0.00001	0.05128	0.556886
145	Ying64	0.00077	0.00555	0.016748	0.022073	0.00001	0.00001	0.087042	0.042282	0.269586	0.555928
146	Zaodahuang	0.019002	0.00001	0.00001	0.038104	0.0234	0.241539	0.166174	0.00001	0.019361	0.49239
147	434	0.052162	0.094418	0.00001	0.09081	0.01833	0.26193	0.023761	0.00001	0.01891	0.43966
148	4112	0.27493	0.021307	0.327544	0.00001	0.189648	0.00001	0.00001	0.00001	0.12575	0.060781
149	7327	0.01924	0.00001	0.00001	0.09445	0.362998	0.00001	0.02696	0.00001	0.00001	0.496301
150	3H_2	0.00001	0.00001	0.472804	0.00001	0.00001	0.527116	0.00001	0.00001	0.00001	0.00001
151	698_3	0.290458	0.00001	0.00001	0.00001	0.103966	0.11305	0.00001	0.00001	0.24725	0.245226
152	75_1	0.014631	0.012235	0.00001	0.00001	0.00001	0.00001	0.264903	0.039591	0.032929	0.635671
153	H152	0.000016	0.00001	0.00001	0.00001	0.393974	0.182153	0.423797	0.00001	0.00001	0.00001
154	Shuang741	0.00001	0.02632	0.00001	0.011002	0.00001	0.202851	0.505233	0.00001	0.033423	0.22113
155	Ye8001	0.005777	0.033641	0.004282	0.085935	0.135875	0.041934	0.021406	0.047608	0.00001	0.623533
156	Yuanqi123	0.00001	0.00001	0.038301	0.00001	0.004836	0.235265	0.14291	0.00001	0.003794	0.574853
157	Chang72	0.00001	0.00001	0.000011	0.00001	0.00001	0.00001	0.383496	0.00001	0.033297	0.583136
158	618	0.00001	0.00001	0.034133	0.038135	0.188608	0.307049	0.064232	0.00001	0.022633	0.345181
159	9058	0.238693	0.00001	0.255559	0.00001	0.045146	0.00001	0.00001	0.00001	0.2872	0.173351
160	Ay420	0.009524	0.028414	0.597908	0.00001	0.00001	0.00001	0.36409	0.000014	0.00001	0.00001
161	DH65232	0.07926	0.019729	0.291706	0.028101	0.031164	0.00001	0.006076	0.021516	0.0715	0.450936

162	912	0.00001	0.00001	0.00001	0.071065	0.078717	0.118518	0.000011	0.731639	0.00001	0.00001
163	3IBZ2	0.00001	0.00001	0.00001	0.00001	0.082402	0.00001	0.00001	0.632955	0.00001	0.284573
164	BCC03	0.00001	0.009648	0.00001	0.029363	0.608145	0.00001	0.00001	0.020111	0.00001	0.332682
165	CS608	0.7306	0.26932	0.00001	0.00001	0.00001	0.00001	0.00001	0.00001	0.00001	0.00001
166	E8501	0.00001	0.063528	0.000014	0.181335	0.00001	0.038677	0.000012	0.716394	0.00001	0.00001
167	F42	0.99991	0.00001	0.00001	0.00001	0.00001	0.00001	0.00001	0.00001	0.00001	0.00001
168	ICI_740	0.99991	0.00001	0.00001	0.00001	0.00001	0.00001	0.00001	0.00001	0.00001	0.00001
169	LH128	0.00001	0.00001	0.00001	0.001467	0.84504	0.00001	0.00001	0.00001	0.00001	0.153422
170	LH160	0.00001	0.00001	0.005342	0.146263	0.074907	0.120134	0.00001	0.65207	0.00001	0.001244
171	LH191	0.077599	0.23815	0.00001	0.00001	0.00001	0.00001	0.00001	0.630739	0.053451	0.00001
172	LH193	0.99991	0.00001	0.00001	0.00001	0.00001	0.00001	0.00001	0.00001	0.00001	0.00001
173	LH194	0.99991	0.00001	0.00001	0.00001	0.00001	0.00001	0.00001	0.00001	0.00001	0.00001
174	LH195	0.99991	0.00001	0.00001	0.00001	0.00001	0.00001	0.00001	0.00001	0.00001	0.00001
175	LH202	0.99991	0.00001	0.00001	0.00001	0.00001	0.00001	0.00001	0.00001	0.00001	0.00001
176	LH205	0.727305	0.272615	0.00001	0.00001	0.00001	0.00001	0.00001	0.00001	0.00001	0.00001
177	LH212Ht	0.015769	0.041674	0.00001	0.00001	0.620533	0.321963	0.00001	0.00001	0.00001	0.00001
178	LH214	0.00001	0.00001	0.00001	0.00001	0.87222	0.1277	0.00001	0.00001	0.00001	0.00001
179	LH215	0.000016	0.00001	0.00001	0.051312	0.678267	0.156397	0.000014	0.075766	0.00001	0.038199
180	LH220Ht	0.312432	0.687488	0.00001	0.00001	0.00001	0.00001	0.00001	0.00001	0.00001	0.00001
181	Lp215D	0.00001	0.00001	0.00001	0.00001	0.668929	0.330991	0.00001	0.00001	0.00001	0.00001
182	MBUB	0.00001	0.00001	0.0113	0.068125	0.106591	0.709579	0.00001	0.00001	0.00001	0.104355
183	ML606	0.00001	0.00001	0.00001	0.006502	0.00001	0.993418	0.00001	0.00001	0.00001	0.00001
184	MQ305	0.00001	0.270617	0.00001	0.00001	0.00001	0.00001	0.00001	0.729295	0.00001	0.000017
185	OS602	0.00001	0.3314	0.00001	0.00001	0.00001	0.000016	0.00001	0.668507	0.000016	0.00001
186	PHP76	0.00001	0.00001	0.00001	0.00001	0.99991	0.00001	0.00001	0.00001	0.00001	0.00001
187	PHP85	0.286383	0.713537	0.00001	0.00001	0.00001	0.00001	0.00001	0.00001	0.00001	0.00001
188	A554	0.02169	0.00001	0.00001	0.070851	0.083288	0.00001	0.00001	0.077232	0.00001	0.746889
189	B95	0.00001	0.186725	0.078943	0.00001	0.163279	0.00001	0.570993	0.00001	0.00001	0.00001
190	CM37	0.066699	0.00001	0.00001	0.011969	0.00001	0.041966	0.00001	0.00001	0.00001	0.879307
191	CM7	0.066483	0.00001	0.00001	0.011204	0.00001	0.041809	0.00001	0.00001	0.00001	0.880445
192	Mo24W	0.040926	0.027731	0.046827	0.024129	0.025319	0.030477	0.002068	0.027492	0.024407	0.750624
193	Mt42	0.04411	0.058611	0.00001	0.00001	0.02123	0.020297	0.006622	0.10193	0.00001	0.747172
194	ND255	0.009852	0.070195	0.00001	0.081237	0.019988	0.029515	0.00001	0.013573	0.00001	0.775611
195	R2	0.038592	0.043139	0.00001	0.036846	0.02199	0.00001	0.00001	0.100056	0.00001	0.759336
196	W9	0.048843	0.037569	0.037846	0.00001	0.01787	0.034232	0.00001	0.029604	0.00001	0.794006
197	A71	0.085745	0.00001	0.035942	0.082338	0.001792	0.002027	0.00001	0.046023	0.00001	0.746102
198	KY228	0.066509	0.036187	0.00001	0.00001	0.066134	0.019717	0.006635	0.057367	0.009998	0.737432
199	B57	0.001722	0.038275	0.039076	0.079999	0.022589	0.021031	0.005504	0.055346	0.00001	0.736447
200	K4	0.00001	0.088899	0.017881	0.043062	0.063398	0.00001	0.00001	0.021471	0.030016	0.735243
201	W23	0.065432	0.043452	0.017503	0.017551	0.021264	0.036211	0.009437	0.04702	0.014487	0.727643
202	K64	0.004753	0.066592	0.065232	0.057868	0.035575	0.008892	0.00001	0.034007	0.00001	0.727061
203	PHH93	0.00001	0.015602	0.061027	0.018707	0.030733	0.009764	0.03176	0.024325	0.084672	0.723399
204	A334	0.018534	0.069617	0.00001	0.00001	0.055653	0.026585	0.008846	0.098361	0.00001	0.722374
205	C46	0.02976	0.056328	0.00001	0.049305	0.035218	0.049268	0.011829	0.048323	0.00001	0.719948

206	A374	0.049997	0.027907	0.024609	0.168424	0.00001	0.01648	0.00001	0.00001	0.00001	0.712543
207	R4	0.050636	0.00001	0.00001	0.100168	0.027132	0.061028	0.00001	0.053852	0.00001	0.707144
208	PHGG7	0.00001	0.037875	0.00001	0.140024	0.031956	0.050835	0.029975	0.022667	0.00001	0.686639
209	H49	0.055679	0.009844	0.00001	0.224985	0.028508	0.00001	0.00001	0.00001	0.00001	0.680934
210	A171	0.00001	0.062812	0.005763	0.069583	0.002998	0.092603	0.015727	0.071086	0.00001	0.679408
211	MS153	0.042581	0.018307	0.021797	0.087844	0.011384	0.110334	0.00001	0.031323	0.00001	0.676412
212	N6	0.021484	0.055871	0.00001	0.066439	0.057938	0.047089	0.00001	0.076586	0.00001	0.674563
213	B8	0.00001	0.125812	0.00001	0.017168	0.039474	0.031413	0.00001	0.117563	0.00001	0.668529
214	H5	0.051693	0.00001	0.024016	0.203509	0.00001	0.019828	0.00001	0.033289	0.00001	0.667625
215	C102	0.00001	0.038457	0.00001	0.141473	0.116409	0.039976	0.00271	0.00001	0.00001	0.660935
216	A375	0.06397	0.053393	0.00001	0.109225	0.00001	0.105525	0.00001	0.007549	0.00001	0.660298
217	WF9	0.02715	0.001454	0.00001	0.264879	0.05043	0.00001	0.00001	0.00001	0.00001	0.656037
218	R168	0.00001	0.121173	0.00001	0.123614	0.00564	0.075794	0.00001	0.021281	0.000012	0.652456
219	A659	0.015269	0.091525	0.00001	0.058295	0.041599	0.12539	0.00001	0.018031	0.00001	0.64986
220	B98	0.031871	0.075321	0.017851	0.051834	0.058145	0.037116	0.003178	0.098466	0.00001	0.626209
221	38_11	0.087348	0.024764	0.00001	0.142578	0.01874	0.017739	0.00001	0.085001	0.006888	0.616921
222	Oh7	0.018344	0.238737	0.002093	0.068271	0.001567	0.00001	0.00001	0.055115	0.00001	0.615843
223	W22	0.027276	0.084702	0.015429	0.138406	0.038877	0.068691	0.00001	0.016886	0.00001	0.609712
224	OH7B	0.043517	0.187586	0.00001	0.093429	0.00001	0.003885	0.00001	0.066191	0.00001	0.605352
225	B96	0.00001	0.17629	0.034042	0.00001	0.00001	0.00001	0.140918	0.00001	0.080831	0.567869
226	ZS01250	0.01782	0.00001	0.00001	0.00001	0.396187	0.00001	0.00001	0.039854	0.00001	0.546078
227	B12	0.005134	0.00001	0.021821	0.00001	0.005678	0.106763	0.277771	0.00001	0.044014	0.538789
228	OQ101	0.070398	0.192826	0.00001	0.089931	0.081601	0.014949	0.000017	0.016147	0.007481	0.526639
229	LH190	0.020312	0.002229	0.00001	0.108084	0.221461	0.06029	0.00001	0.073326	0.00001	0.514268
230	904	0.053253	0.00001	0.00001	0.000016	0.37768	0.00001	0.00001	0.056935	0.00001	0.512066
231	LH162	0.014435	0.044302	0.00001	0.00001	0.397681	0.00001	0.00001	0.062429	0.00001	0.481103
232	I_205	0.078925	0.065396	0.00001	0.037583	0.058305	0.00001	0.00001	0.30788	0.00001	0.451872
233	B10	0.145491	0.283608	0.00001	0.00001	0.00001	0.00001	0.00001	0.123818	0.00001	0.447023
234	M14	0.066971	0.043777	0.00001	0.194863	0.00001	0.192679	0.00001	0.055158	0.00001	0.446511
235	2MA22	0.00001	0.04043	0.015198	0.164593	0.063243	0.267026	0.00001	0.025499	0.00001	0.423981
236	RS_710	0.00001	0.447871	0.00001	0.042116	0.002623	0.062361	0.00001	0.026021	0.00001	0.418968
237	PHW30	0.026924	0.009747	0.001758	0.00001	0.534446	0.00001	0.00001	0.023801	0.00001	0.403284
238	H99	0.00001	0.00001	0.00001	0.068011	0.00001	0.522217	0.00001	0.023208	0.00001	0.386503
239	6M502A	0.00001	0.045062	0.044956	0.143922	0.225086	0.191993	0.00001	0.00001	0.000302	0.348649
240	R177	0.243403	0.054292	0.00001	0.065466	0.017558	0.205326	0.00001	0.078694	0.00001	0.335231
241	N28	0.314949	0.229037	0.00001	0.000012	0.053437	0.016818	0.00001	0.063922	0.00001	0.321795
242	PHM81	0.011852	0.169844	0.00001	0.026981	0.00001	0.00001	0.00001	0.498767	0.00001	0.292506
243	B100	0.014365	0.000012	0.00001	0.094107	0.00001	0.586218	0.00001	0.017546	0.00001	0.287712
244	F118	0.6278	0.060211	0.011709	0.00001	0.014117	0.00001	0.00001	0.00001	0.00001	0.286114
245	CI_187_2	0.143826	0.00001	0.00001	0.00001	0.479469	0.00001	0.00001	0.092833	0.00001	0.283813
246	LH163	0.043677	0.263538	0.00001	0.288859	0.00001	0.00001	0.00001	0.110511	0.012228	0.281148
247	H84	0.17376	0.350444	0.00001	0.08236	0.00001	0.071301	0.00001	0.00001	0.081432	0.240663
248	C123	0.00001	0.015146	0.02686	0.108572	0.58536	0.00001	0.00001	0.00001	0.026017	0.238005
249	PHR55	0.00001	0.00001	0.023089	0.567367	0.035588	0.00001	0.00001	0.107415	0.04989	0.216612

250	PHR30	0.015034	0.371276	0.00001	0.148591	0.141694	0.126226	0.00001	0.016941	0.001111	0.179108
251	FBLA	0.147228	0.613423	0.00001	0.00001	0.00001	0.032857	0.00001	0.040728	0.00001	0.165714
252	PHV53	0.00001	0.011158	0.035276	0.55495	0.00001	0.149778	0.00001	0.00001	0.097923	0.150875
253	PHW43	0.025504	0.00001	0.033671	0.212587	0.013501	0.233931	0.00001	0.269884	0.06917	0.141732
254	2FACC	0.588122	0.237564	0.00001	0.036047	0.002744	0.003039	0.00001	0.00001	0.00001	0.132445
255	PHVA9	0.288351	0.307426	0.019439	0.112728	0.074329	0.045508	0.00001	0.034456	0.010486	0.107267
256	PHJ40	0.061466	0.201814	0.00001	0.067075	0.428646	0.0002	0.00001	0.001285	0.142583	0.096912
257	6F629	0.346508	0.504766	0.000017	0.00001	0.009407	0.00001	0.00001	0.00134	0.050995	0.086938
258	CS405	0.584855	0.19229	0.00001	0.033993	0.00001	0.00001	0.00001	0.025645	0.082468	0.08071
259	PHN66	0.00001	0.731604	0.017794	0.00001	0.148768	0.00001	0.00001	0.00001	0.021373	0.08041
260	LH192	0.936758	0.00001	0.00001	0.00001	0.00001	0.00001	0.00001	0.00001	0.00001	0.063162
261	PHJ89	0.00001	0.008398	0.00001	0.479559	0.133394	0.317395	0.00001	0.00001	0.00001	0.061204
262	LH222	0.558096	0.387908	0.00001	0.007151	0.00001	0.00001	0.00001	0.001564	0.00001	0.045231
263	PHPR5	0.143879	0.581704	0.018418	0.030242	0.16886	0.026465	0.000011	0.00001	0.00001	0.030401
264	PHG35	0.085759	0.266226	0.003333	0.416944	0.069281	0.124559	0.00001	0.00001	0.015841	0.018038
265	ICI_893	0.880763	0.00001	0.00001	0.042024	0.004714	0.068787	0.00001	0.00001	0.00001	0.003663
266	LH209	0.845715	0.154193	0.00001	0.00001	0.00001	0.000011	0.000014	0.00001	0.00001	0.000017
267	787	0.918549	0.081368	0.00001	0.00001	0.00001	0.00001	0.00001	0.00001	0.00001	0.000013
268	NS501	0.00001	0.501353	0.00001	0.00001	0.00001	0.00001	0.00001	0.498567	0.00001	0.00001
269	ICI_193	0.130408	0.413189	0.00001	0.00001	0.00001	0.00001	0.00001	0.456333	0.00001	0.00001
270	LH208	0.560504	0.408207	0.00001	0.019386	0.00001	0.00001	0.00001	0.011842	0.00001	0.00001
271	NL001	0.735805	0.264114	0.00001	0.00001	0.00001	0.00001	0.00001	0.000011	0.00001	0.00001
272	PHT55	0.00001	0.956425	0.00001	0.00001	0.00001	0.00001	0.00001	0.00001	0.043495	0.00001
273	IBB14	0.724483	0.244875	0.005651	0.00001	0.001887	0.00001	0.00001	0.00001	0.023054	0.00001
274	LH196	0.999909	0.00001	0.00001	0.00001	0.00001	0.00001	0.00001	0.00001	0.000011	0.00001
275	PHW51	0.00001	0.964332	0.000013	0.00001	0.035585	0.00001	0.00001	0.00001	0.00001	0.00001
276	ICI_441	0.999908	0.00001	0.00001	0.000012	0.00001	0.00001	0.00001	0.00001	0.00001	0.00001
277	LH206	0.748273	0.251644	0.00001	0.000013	0.00001	0.00001	0.00001	0.00001	0.00001	0.00001
278	CI31A	0.011628	0.038225	0.017484	0.060108	0.016916	0.035778	0.00001	0.105513	0.040026	0.674314

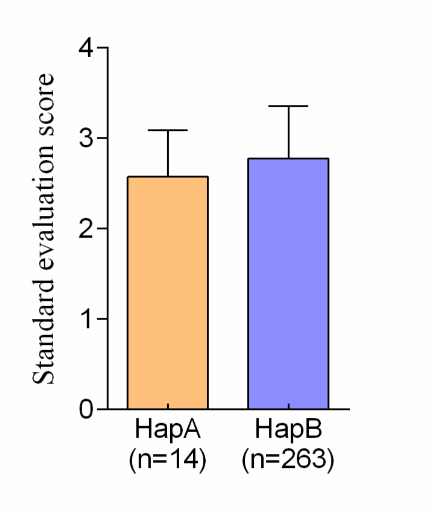

Figure S1. The standard evaluation score (SES) of HapA and HapB

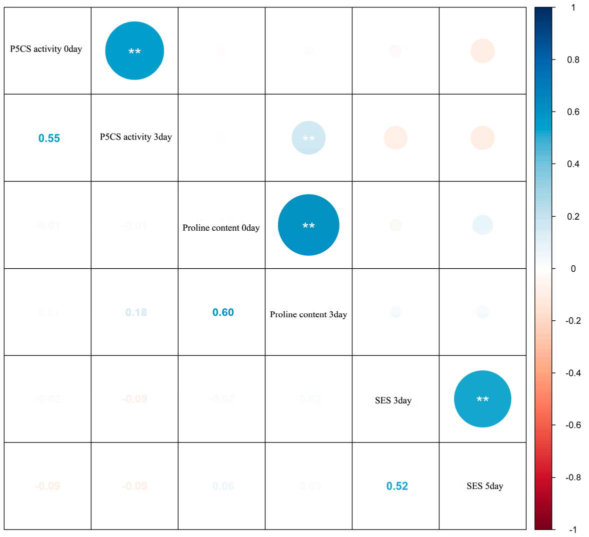

Figure S2. Relationship among P5CS activity, Proline content, and Standard evaluation score
